# The impact of Chinese volume-based procurement on pharmaceutical market concentration

**DOI:** 10.3389/fphar.2024.1386533

**Published:** 2024-06-04

**Authors:** Ying Yang, Yuxin Liu, Zongfu Mao, Jing Mao, Yalei Jin

**Affiliations:** ^1^ School of Nursing, Tongji Medical College, Huazhong University of Science and Technology, Wuhan, China; ^2^ Global Health Institute, Wuhan University, Wuhan, China; ^3^ Dong Fureng Institute of Economic and Social Development, Wuhan University, Wuhan, China; ^4^ Tongji Hospital, Tongji Medical College, Huazhong University of Science and Technology, Wuhan, China; ^5^ Department of General Practice, Zhongnan Hospital of Wuhan University, Wuhan University, Wuhan, China

**Keywords:** volume-based procurement, group purchasing, pharmaceutical industry, market concentration, drug market

## Abstract

**Objectives:**

Optimizing the pharmaceutical industrial structure is the key mission of China’s healthcare reform. From the industrial structure perspective, this study empirically evaluated the impact of China’s national volume-based procurement (NVBP) policy on market concentration in the hospital-end drug market.

**Methods:**

This study used drug procurement data of China’s public medical institutions which obtained from the national database. A quasi-natural experiment was designed involving eleven pairs of matched treatment-control region combinations, with NVBP policy as the intervention measure. The market was defined by drug name (molecular boundary) and city/province (geographical boundary). Market changes were measured from three dimensions: the number of enterprises and products, market share, and Herfindahl-Hirschman index (HHI). Dual comparison approach and difference-in-difference (DID) method with fixed effect model were applied to quantify policy impacts.

**Results:**

The number of enterprises and products decreased by 18 and 83 in pilot regions after NVBP policy, far more than the decreases in control regions (6 and 21). The accumulative market share of 15 bid-winning enterprises increased by 53.67% in volume and 18.79% in value, among which the increment of enterprises with low baseline market share was more prominent (66.64% and 36.40%). Among three enterprise types, the market share of generic consistency evaluation (GCE) certificated generics significantly increased, GCE uncertificated generics significantly decreased, and originators slightly decreased. DID models indicated significantly positive impact of NVBP policy on market concentration, with HHI-volume and HHI-value increasing by 49.33% (*β* = 0.401, *p* < 0.01) and 21.05% (*β* = 0.191, *p* < 0.01).

**Conclusion:**

The implementation of NVBP promoted the intensive drug circulation and supply of Chinese public hospitals, intensifying the exit of GCE uncertificated generics from the hospital-end market. NVBP combined with GCE standards significantly improved market concentration, which brought a positive signal of pharmaceutical industrial structure optimization in China. In the future context of normalized and institutionalized NVBP, the balance should be further sought between low drug prices and reliable hospital drug supply, sustainable industry development.

## 1 Background

Pharmaceutical industry, a strategic industry related to the health of the whole people, it’s development determines whether people can obtain high-quality drug supply timely. Industry structure is the most important determinant of industrial performance (George et al., 2005), and in general, the improving of supply-side concentration is conducive to better resource allocation efficiency for a originally decentralized competitive market (Wei, 2003). In China, supply-side structural reform has always been the main line of the whole medical and healthcare system reform (Shen and Zhang, 2017; Ding, 2019).

China’s pharmaceutical industry has long been a “many, scattered, and small” situation. Statistics show that, by the end of 2018, the number of pharmaceutical wholesale enterprises in China reached 13,600 (Deng and Wen, 2019), in which the figure of large-scale manufacturing enterprises^1^ was only 7,581 (National Bureau of Statistics, 2019). The market share of top 100, 50, and 10 pharmaceutical manufacturing enterprises were only 32.5%, 25.5%, and 11.0% in China (Insight and Info, 2019). In stark contrast, the top 3 enterprises in the United States and top 5 in Japan has already possessed 90% and 75.9% market share (Shi et al., 2022). The reason of low pharmaceutical industrial concentration in China, on the one hand, is the absence of policy environment to guide the fair competition between generic and originator drug manufacturers in the past; on the other hand, domestic enterprises are generally small-scaled and low innovated, which is difficult to form effective market competitiveness (Liu et al., 2018; Ding, 2019).

To address the situation of low-end duplication of domestic generic drug manufacturing, the National Medical Products Administration (NMPA) carry out the generic consistency evaluation (GCE) in 2012 (General Office of the State Council, 2012; National Medical Products Administration, 2013) to re-evaluate the marketed domestic generic drugs and ensure their consistency in quality and efficacy with corresponding original drugs. In this way, the quality of generic drugs can be ensured at the source of drug approval, and the structure of the drug manufacturing industry can be optimized (Hu and Yu, 2016). Since 2018, Chinese government has vigorously promoted the National Volume-Based Procurement (NVBP) policy (General Office of the State Council, 2021). The off-patent drugs with generic enterprises had gained GCE certification were selected for the procurement list, a cross-regional procurement alliance was established, and the public medical institutions were mainly involved, for 80% of drugs were consumed there. The policy measures of NVBP brought a signal of “reshuffle” in the pharmaceutical industry: 1) the setting of the threshold for enterprise participation, only the products reached GCE quality standard^2^ and the enterprises met the supply capacity requirement have the chance for participation; 2) the concentration of procurement demand, that is, the procurement volume of all the public medical institutions in all the alliance regions was pooled, and 60%–70% of them was taken as the bidding target, which not only improves the buyer power but also increases the cost of enterprises to abandon the NVBP market; and 3) the strict implementing of “volume-based” procurement, different from the previous procurement mode that highly relies on commercial promotion and illegal kickbacks to ensure drug sales (Mao et al., 2020), once the enterprises win the bid in NVBP, they will get most of the drug market, that is, sufficient sales guarantee. NVBP policy promoted the actual implementation of the GCE standard, and may help to improve the structure of China’s pharmaceutical industry (Li et al., 2012; Wang et al., 2012; Wang, 2015).

Current numerous studies regarding NVBP policy mainly focus on the effect of purchasing consortia on drug price reduction (Wang et al., 2021; Long et al., 2022), drug expenditure cutting (Chen et al., 2020), and drug accessibility improvement (Yuan et al., 2021; Zhao et al., 2022). While, from the perspective of the pharmaceutical industry structure, it’s still unclear the change of market concentration under NVBP implementation, as well as the future trends. Only one study provided inference through theoretical analysis (Huang and Tao, 2020). Given that, from the perspective of industrial structure, this study aim to empirically explore the impact of the first round pilot of NVBP on market concentration in the hospital-end drug market.

## 2 Literature review

Market concentration is the primary determinant of industry structure (George et al., 2005), including two elements—the number of firms and the market share of incumbent firms. We summarized the impact of tendering and procurement policy on three aspects: the number of pharmaceutical enterprises, the market share of different enterprises, and the market concentration.

In Sweden’s practice, a monthly bidding approach was used to determine the sole supplier (the lowest bidder) for each off-patent drug (by substance-strength-form-package size combination), Bergman et al. (2017) found that a 1% increment in the market share of lowest bidder would result in approximately 1% decrease in the number of firms in the market. Similarly, during China’s essential medicine system reform, a single supplier tendering and procurement method was also applied, Barber et al. (2013) pointed out that this approach may exacerbate dependence on specific suppliers. From the perspective of pharmaceutical enterprise collusion, Zhu et al. (2022) constructed a dynamic procurement model and found that the NVBP mechanism has a long-term effect on market structure, which is reflected in decreasing the number of future incumbent enterprises by affecting the current situation of current bid-winners. Tan and Wu (2022), based on Porter’s five forces model and the AHP-SWOT method, noted that the shortage of small and medium-sized enterprises in funding, cognition of policy and market, drug quality and innovation, will put them at a disadvantage position in competition with large-sized enterprises in the context of NVBP mechanism. These studies to some extent explained why tendering and centralized procurement might lead to a decrease in the number of pharmaceutical enterprises.

Existing research regarding the impact of the centralized procurement mechanism on market share mainly focused on two aspects—the bidding attribute (bid-winner or bid-non-winner) and the product attribute (generics or originators). The former, the role of centralized procurement in increasing the market share of bid-winning enterprises is self-evident, which is also the logical underpinning of the centralized procurement mechanism in promoting scale economy (Huff-Rousselle, 2012). Previous empirical studies have revealed this change (Bergman et al., 2017; Lu et al., 2022), with the consumption proportion of bid-winning products increasing from 17.03% to 73.61% after the NVBP policy. The latter, generic enterprises have greater motivation to exchange market share through price reductions, making it a higher probability of winning the bid (Sun et al., 2022). Wang et al. (2022) and Xie et al. (2021) declared a significant increment in the market share of generic enterprises after NVBP policy implementation.

Few studies have directly explored the impact of centralized procurement on market concentration. Wouters et al. (2019) outlined the trends of drug prices and market concentration from 2003 to 2016 under the context of pharmaceutical tendering in South Africa, and found the market concentration of anti-tuberculosis drugs and anti-tumor drugs showed an increasing trend, while that of anti-retroviral drugs and anti-infective drugs is decreasing, which has not yet revealed the consistent law of the impact of bidding on the market concentration. Regarding the implementation of the national essential medicine system in China, Wang et al. (2012) and Li et al. (2012) observed the change in the pharmaceutical market of primary medical institutions in Shanghai, found that the concentration ratio of top 1, 3, and 8 enterprises (CR_1_, CR_3_, and CR_8_) had increased after the reform, and the market share tended to be concentrated towards large foreign-funded enterprises. They believed that this change was associated with the unified bidding and single supply mode of primary medical institutions. Wang (2015) analyzed the drug bidding documents of 15 provinces in China, and found that the provinces that made clear provisions and restrictions on tender’s scale and competitive capability owned higher bid-winner concentration. After the launch of NVBP, Huang and Tao (2020) declared that the policy provided necessary conditions for the improvement of industry concentration through the theoretical discussion of enterprise scale, market capacity, and market entry barriers. However, Li et al. (2023) observed the antibacterial drug market and found that the market share of top 3 enterprises has changed significantly after NVBP policy, but the industry ratio (CR_3_) has not increased.

In summary, the centralized procurement mechanism (especially the single supplier mode) may lead to a decrease in the number of incumbent enterprises and the number of registered products. On the positive side, it may promote the concentration of drug supply; while on the negative side, it may lead to monopolies or supply interruptions (Grootendorst and Hollis, 2012; Dranitsaris et al., 2017). Volume-based procurement is essentially a group purchase by large buyers, which inevitably leads to a tilt in market share towards bid-winning enterprises. Then, under the realistic situation of differences in the bid-winning enterprises type, bid-winning products type, and baseline market competition pattern, what impacts will NVBP policy bring to the market concentration and what are the future market trends? It’s not entirely clear.

## 3 Materials and methods

### 3.1 Data sources

This study used data from the China Drug Supply Information Platform (CDSIP), which covered drug procurement order data of all provincial drug centralized procurement platforms from 31 provinces (autonomous regions and municipalities) in the Chinese mainland. CDSIP, constructed and operated by the Statistical Information Center of the National Health Commission and officially launched in October 2015, is used to obtain information about medical institutions’ daily drug orders, storage, delivery, and settlement. It mainly meets the government’s monitoring and management requirements on drug prices, quantities, distribution, and warehousing information in medical institutions. CDSIP database has the advantages of authenticity, accuracy, and strong representativeness of data. First, the data comes from the original purchasing order information conducted by medical institutions in the corresponding provincial drug procurement platform, which is transmitted in real-time and cannot be tampered with by medical institutions, and is more accurate than hospital reporting data. Second, under the policy constraint that “all drugs prescribed in hospitals (excluding Chinese herbal pieces) should be procured through the provincial drug procurement platform” (General Office of the State Council, 2015), the coverage of medical institutions in the CDSIP database is well-guaranteed, which is estimated to cover more than 80% of the drug procurement information of medical institutions in the Chinese mainland. By the end of 2021, CDSIP has integrated large-scale real-world data containing 7,782 drug international nonpropietary names (INN), 137,646 drug generic names, and 146,993 products underlying drug attribute information and order information. The database covers drug procurement data from 48,205 public medical institutions in 31 provinces, including 9,176 public hospitals and 39,029 primary medical institutions (Yang et al., 2022).

Data extracted from the CDSIP database include the name of the medical institution, procurement date, product YPID (Yao Pin Identifier) code, drug generic name, dosage form, specification, package, manufacturer, unit price, purchasing unit (by box, bottle, or branch), purchase quantity and value. In this study, we collected data on 25 policy-covered INNs (Joint Procurement Office, 2018a; Joint Procurement Office, 2018b) from January 2018 to November 2019. A total of 12 therapeutic categories and 15 bid-winning enterprises were involved in the included analytical drugs, as shown in Supplementary Appendix A.

### 3.2 Measurement

#### 3.2.1 Measure of NVBP policy intervention

This study examines the impact of the first round of the NVBP pilot. Between November 15 and 17 December 2018, the Joint Procurement Office organized and completed the bidding of the first round pilot work, and publicized the information about the bid-winning enterprises and their products. Under the unified requirements of the NHSA, all medical institutions in all pilot cities started implementing the bidding results from March 1 to 1 April 2019, which means purchasing drugs from the bid-winning enterprise at their bidding price (Yang et al., 2021). Therefore, we set the time point of policy intervention as March 2019, that is, January 2018 to February 2019 is the pre-intervention period, and March to November 2019 is the post-intervention period.

The first round of pilot work was implemented in 11 cities, including 4 municipalities (Beijing, Tianjin, Shanghai, and Chongqing) and 7 sub-provincial cities (Shenyang, Dalian, Xiamen, Guangzhou, Shenzhen, Chengdu, and Xi’an) (also known as the “4 + 7” pilot) (General Office of the State Council, 2019), thus we included all 11 cities as the intervention group. At that time, regions except for 11 pilot cities in the Chinese mainland had not yet implemented the NVBP policy, which allowed us to apply quasi-natural experimental design at the regional level. In four steps, regions with relative comparability were selected from the provinces that had not implemented NVBP to constitute the control group: 1) stratification of observation regions; 2) selection of region-level matching variables; 3) TOPSIS stratified matching to determine control regions; 4) comparison of baseline balance between the intervention and control regions. The detailed procedure is outlined in Supplementary Appendix B.

#### 3.2.2 Market-related measurement

The first step in market analysis is to determine market boundaries, including molecular boundaries and geographical boundaries (Goodman et al., 2009). First, in the field of off-patent drugs, many manufacturers engage in generics production after the expiration of drug patents, and the product competition is mainly reflected among manufacturers with the same effective ingredient and the same curative effect. Thus, we take INN as the molecular boundary. Second, drug bidding and procurement is mainly carried out by provincial units in the Chinese mainland, and there are significant differences in the drug market among provinces (Shi et al., 2020). Therefore, we set province/city as the geographical boundary.

Market changes brought by NVBP policy are measured from three dimensions—the number of enterprises and products, market share, and market concentration. 1) The number of products and pharmaceutical enterprises of specific drugs with sale records in observation regions during the observation period. The product is defined according to the unique identification code—YPID. 2) Two aspects of market share were observed: the bidding attribute (bid-winner or bid-non-winner) and the product attribute (generics or originators). The former refers to the proportion of bid-winning enterprise’s drug sales in volume or value; the latter refers to the constituent ratio in volume or value between generic and original enterprises. Considering the non-summability of quantity among different drugs, this study standardizes the sales quantity using the DDD (Defined Daily Dosage) method recommended by the WHO Collaborating Centre for Drug Statistics Methodology (2023), that is, the quantity of market sales of specific drugs is represented by DDDs. 3) Herfindahl-Hirschman index (HHI) was calculated to measure market concentration, including HHI in volume (HHI-volume) and HHI in value (HHI-value). The calculation of HHI is as follows:

$$\text{HHI}=10000*\sum_{i=1}^{n}{\left(X_{i}/X\right)}^{2}$$ (1)

where *n* is the number of enterprises, *X* represents the total market size, which is the cumulative sales volume or value, *X*_*i*_ is the sales volume or value of the enterprise *i*, and *X*_*i*_/*X* refers to the market share in volume or value of the enterprise *i*. The results of HHI range from 0 to 10,000, and a higher HHI indicates a higher market concentration (U.S. Department of Justice, 2018).

### 3.3 Data analysis

In this study, the dual comparison method (pre-NVBP vs. post-NVBP, pilot regions vs. control regions) and descriptive statistical method, pared-samples *t*-test were adopted to quantify and visualize market changes. Under the quasi-natural experiment framework, the difference-in-difference (DID) model was applied to estimate the impact of NVBP implementation on market concentration, as follows:

$$Y_{\text{ijt}}=\alpha +\beta D_{\text{it}}+\gamma X_{i}+\mu_{i}+\theta_{t}+\delta_{j}+\varepsilon_{\text{ijt}}$$ (2)

where, *Y*_*ijt*_ refers to market concentration of INN *j* in region *i* during month *t*, which is converted to logarithmic form. *D*_*it*_ is a dummy variable for policy intervention, which is coded 1 if NVBP policy is implemented in region *i* during month *t*, otherwise coded 0. The coefficient *β* of *D*_*it*_ term represents the “net effect” of policy impact. *X*_*i*_ is a series of covariates, including three district-level control variables referring to relevant empirical studies (Dubois et al., 2021; Li et al., 2021; Wang and Zahur, 2022), that is, per capita gross domestic product (GDP), population, and per 10,000 population medical institution beds. The covariates were converted logarithmically and denoted as *lngdp*, *lnpop.*, and *lnbeds*. Relevant census data for calculating *X*_*i*_ were collected from the China Statistical Yearbook (National Bureau of Statistics, 2020). Fixed effects were applied to control the potential impact of invisible factors, including the region-fixed effect (*μ*_*i*_), time-fixed effect (*θ*_*t*_), and drug-fixed effect (*δ*_*j*_). *ε*_*ijt*_ refers to the random error term. A common pre-trend test was used to verify the premise of DID method to identify causal effects.

## 4 Results

Twenty-five NVBP policy-covered INNs were included in the analysis, involving a total of 69 generic names, 905 products, and 214 pharmaceutical enterprises. The cumulative market size of these drugs reached 11.28 billion DDD and 63.51 billion CNY in the observation regions, in which the values were 5.50 billion DDD and 28.57 billion CNY in pilot regions and 5.78 billion DDD and 34.94 billion CNY in the control regions.

### 4.1 The number of enterprises and products

As shown in Table 1, the total number of enterprises in non-pilot regions remained unchanged (184 vs. 185) before and after NVBP policy implementation, contrast that, pilot regions decreased by 22 (193 vs. 171), in which the primary contributor was amlodipine (descending from 58 to 40). After the NVBP policy, the number of products decreased by 83 (708 vs. 625) in the pilot group, significantly higher than the decreased value in the control group (21). In pilot regions, 16 out of 25 INNs were observed with product numbers decreasing, including 32 for amlodipine, 11 for cefuroxime, and 8 for montmorillonite.

**TABLE 1:** Descriptive changes in the number of enterprises and products under NVBP policy intervention.

No.	INN	Pilot regions		Control regions	
		Pre-NVBP	Post-NVBP	Pre-NVBP	Post-NVBP
1	Amlodipine	58/123	40/91	42/94	37/77
2	Losartan	12/31	11/28	12/28	11/28
3	Irbesartan	17/45	16/37	17/46	17/40
4	Irbesartan and Hydrochlorothiazide	7/22	7/22	9/25	7/22
5	Fosinopril	2/5	2/4	2/4	2/3
6	Lisinopril	9/13	9/11	10/17	7/10
7	Enalapril	16/41	16/37	21/53	20/48
8	Atorvastatin	8/35	9/33	8/24	10/26
9	Rosuvastatin	8/37	8/37	8/43	8/43
10	Levetiracetam	6/19	6/15	6/15	7/18
11	Olanzapine	5/18	7/19	5/18	7/24
12	Risperidone	11/35	12/35	11/34	11/34
13	Dexmedetomidine	6/23	6/21	5/16	6/21
14	Escitalopram	6/22	6/22	6/19	6/21
15	Paroxetine	5/11	5/12	5/10	5/9
16	Gefitinib	2/3	3/5	2/3	3/4
17	Imatinib	4/9	4/8	4/7	5/8
18	Pemetrexed	13/37	11/27	11/32	12/30
19	Cefuroxime	20/64	18/53	20/81	20/77
20	Entecavir	12/30	12/28	12/37	13/39
21	Tenofovir Disoproxil	6/12	6/11	6/11	7/11
22	Montmorillonite	23/39	17/31	23/36	24/37
23	Clopidogrel	3/10	3/9	3/10	3/10
24	Flurbiprofen	2/4	2/4	2/4	2/4
25	Montelukast	5/20	6/25	5/22	6/24
	Total	193/708	171/625	184/689	185/668

Note: The value in the table refers to “the number of enterprises/the number of products.” INN, international nonpropietary name; NVBP, national volume-based procurement.

### 4.2 Market share

#### 4.2.1 Market share of bid-winning enterprises

We compared the market share of bid-winning enterprises before and after the NVBP policy. In pilot regions, the market share of 15 bid-winning enterprises sharply increased 53.67% (from 21.51% to 75.19%) in volume share and 18.79% (from 29.91% to 48.71%) in value share, in contrast, this figure generally remained stable in non-pilot regions. There were 3 exceptions out of 25 INNs, among them the market share of bid-winning enterprises did not increase in pilot regions. That is, Gefitinib produced by AstraZeneca decreased by 1.78% in market share in value, Imatinib produced by Hansoh Pharma decreased by 1.34% in market share in value, Flurbiprofen produced by Tide Pharmaceutical decreased by 2.81% in market share in volume and 3.53% in market share in value. The results of paired sample *t*-test showed that, in pilot regions, the volume share and value share of bid-winning enterprises after NVBP policy were significantly higher than those before the policy, with the differences of 41.99 ± 25.67 (*t* = 8.17, *p* < 0.001) and 23.13 ± 21.29 (*t* = 5.43, *p* < 0.001), respectively; in the non-pilot regions, no significant difference in volume share (*t* = −0.72, *p* = 0.481) and value share (*t* = 2.44, *p* = 0.224) of bid-winning enterprises was observed after NVBP policy. Data of each INNs are detailed in Supplementary Appendix C.

**FIGURE 1:**
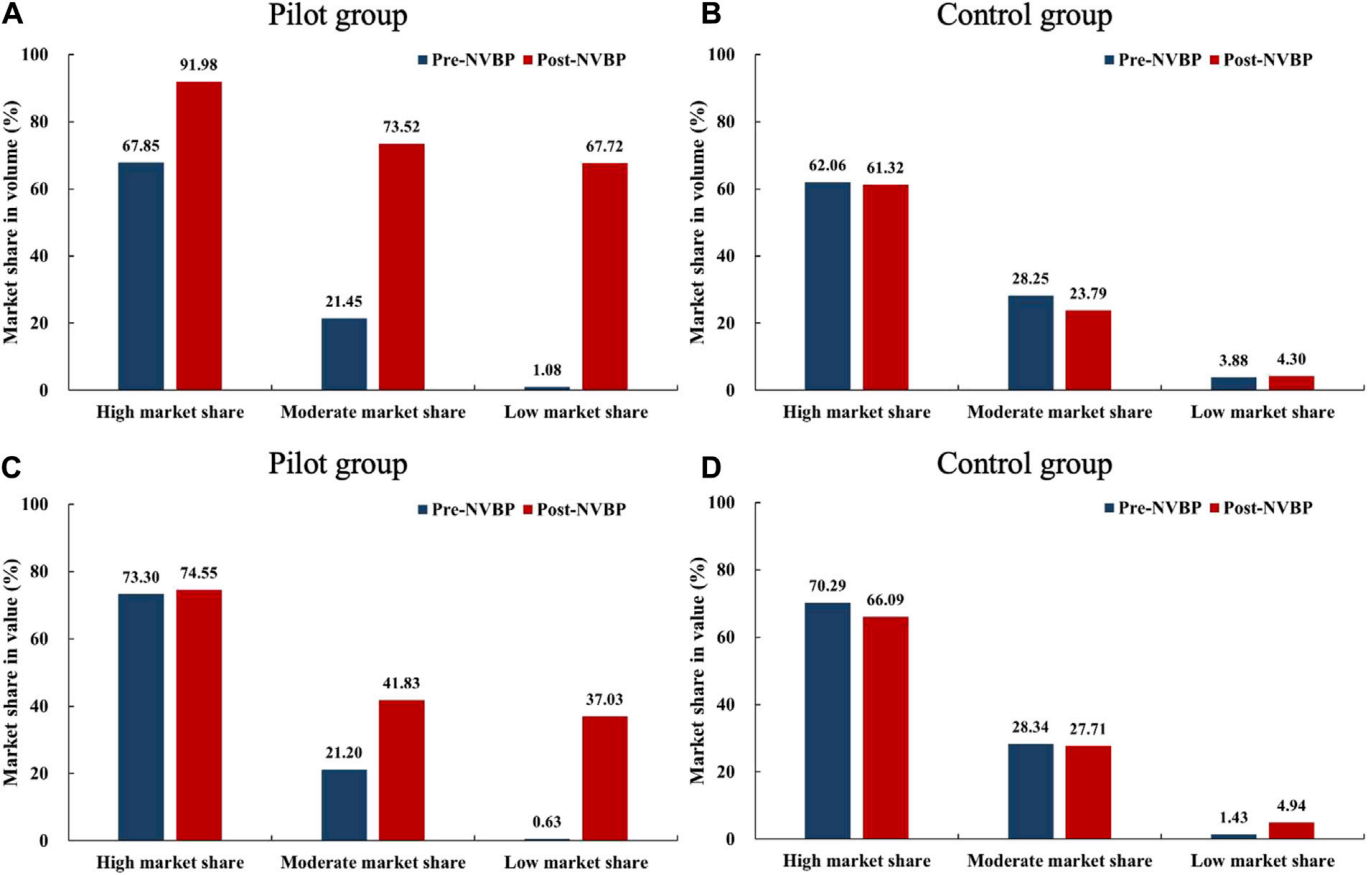
Changes in the market share (in volume and value) of bid-winning enterprises after NVBP implementation by baseline market share levels. **(A)** Market share in volume of pilot group, **(B)** market share in volume of control group, **(C)** market share in value of pilot group, **(D)** market share in value of control group. Note: NVBP, national volume-based procurement.

We divided 25 drugs into three groups based on the baseline (pre-NVBP period) market share of bid-winning enterprises: a) high market share group (*n* = 8), the volume or value share of bid-winner exceeds 50%; b) low market share group (*n* = 8): the volume and value share of bid-winner are less than 10%; c) medium market share group (*n* = 9), other drugs excluding the above two groups. As shown in Figure 1, in the high market share group, the bid-winners’ volume share increased to over 90% in pilot regions after the NVBP policy (67.85% vs. 91.98%), while the value share remained unchanged (70.30% vs. 74.55%). Among these drugs, the bid-winning enterprises almost achieved a monopoly on the volume share of hospital-end market after policy intervention. In low and medium market share groups, bid-winners’ market share, both in volume and value, improved markedly after NVBP implementation in pilot regions, and the growth in volume share was far more prominent than the value share. The low market share group was observed with an increase of 66.64% (from 1.08% to 67.72%) in volume share and 36.40% (from 0.63% to 37.03%) in value share.

**FIGURE 2:**
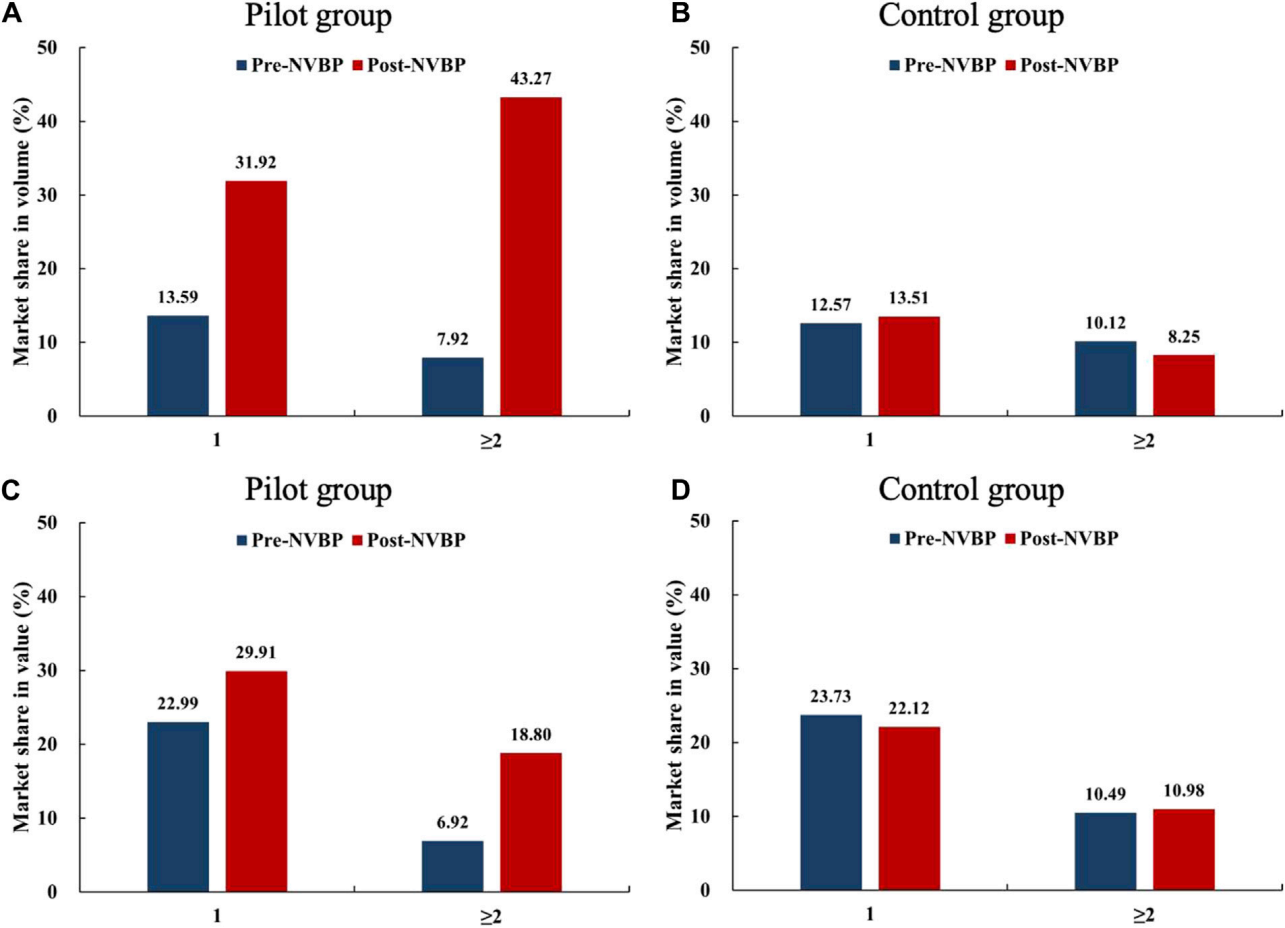
Changes in the market share (in volume and value) of bid-winning enterprises after NVBP implementation by the number of drugs won. **(A)** Market share in volume of pilot group, **(B)** market share in volume of control group, **(C)** market share in value of pilot group, **(D)** market share in value of control group. Note: NVBP, national volume-based procurement.

We further classified 15 enterprises into two groups: a) enterprises that only won one INN (*n* = 10) and enterprises that won two or more INNs (*n* = 5). As shown in Figure 2, enterprises that won ≥2 drugs were observed with higher growth in both market share in volume and in value in the pilot regions than that only won one drug. After NVBP policy, the increment in bid-winning enterprises’ volume share was far higher than the value share, which is more prominent among enterprises that have won ≥2 drugs.

#### 4.2.2 Market share between originator and generic enterprises

**FIGURE 3:**
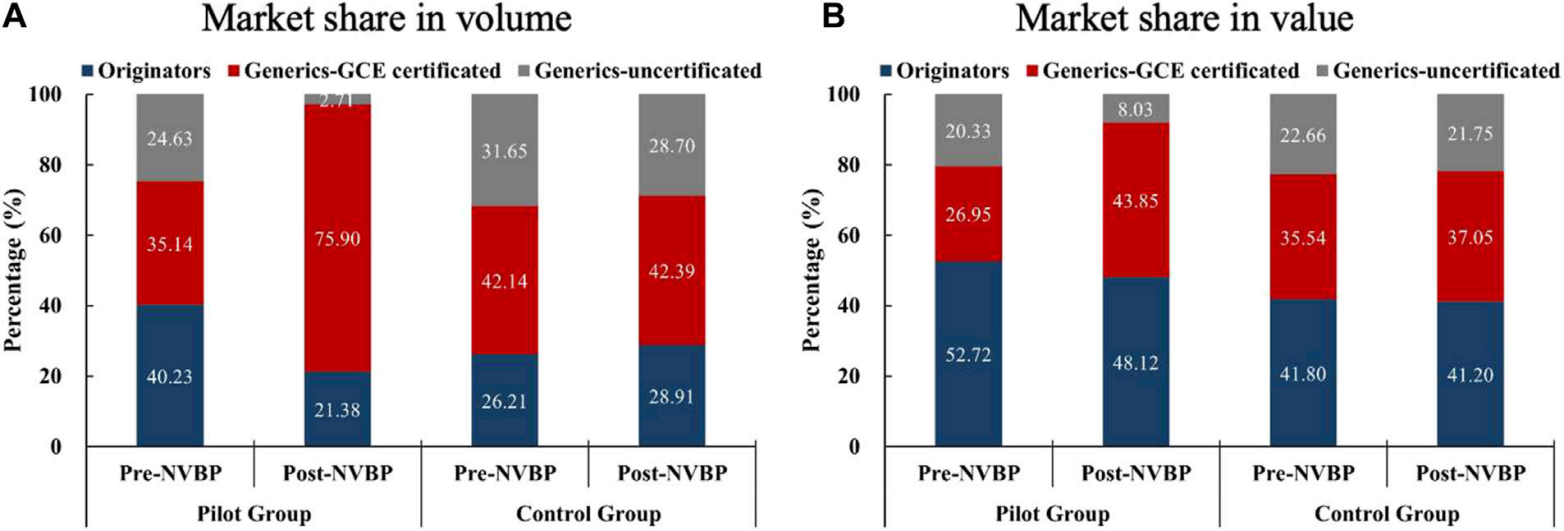
Changes in the market share (in volume and value) among three product groups (originators, GCE certificated generics, and GCE uncertificated generics) after NVBP implementation. **(A)** Market share in volume, **(B)** market share in value. Note: GCE, generic consistency evaluation; NVBP, national volume-based procurement.

Included drugs were dichotomized into originator and generics, further, generics were divided into GCE certificated and uncertificated drugs based on the GCE status as of the end of March 2019. As shown in Figure 3, in non-pilot regions, the market share among three product groups (originators, GCE certificated generics, and GCE uncertificated generics) generally remined stable under NVBP intervention, in which GCE uncertificated generics accounted for about 30% of the volume share and 20% of the value share. In contrast, the market share among three groups in pilot regions demonstrated the characteristic of “one upward and two downwards” after NVBP policy: GCE certificated generics exhibited great increment of 40.76% (from 35.14% to 75.90%) in volume share and 16.90% (from 26.95% to 43.85%) in value share; originators showed an evident decrease of 18.85% (from 40.23% to 21.38%) in volume share and slightly decrease of 4.60% (from 52.72% to 48.12%); GCE uncertificated generics observed sharply decline in market share in both volume (21.92% decline) and value (12.30% decline), which only accounts for 2.71% of the volume share and 8.03% of the value share after NVBP implementation. The detailed market share changes for each included INNs were presented in Supplementary Appendix C.

The results of paired sample *t*-test showed that, in pilot regions, the volume share and value share of GCE certificated generics significantly increased after NVBP policy, with the differences of 36.38 ± 26.58 (*t* = 6.84, *p* < 0.001) and 20.53 ± 23.70 (*t* = 4.33, *p* < 0.001); the volume share of originators significantly decreased by 12.80 ± 16.36 (*t* = −3.67, *p* = 0.001) while the change of value share of originators had no significance (*t* = −1.09, *p* = 0.288); the volume share and value share of GCE uncertificated generics significantly decreased after NVBP policy, with the differences of −29.89 ± 23.75 (*t* = −5.77, *p* < 0.001) and −20.73 ± 20.21 (*t* = −4.70, *p* < 0.001). In the non-pilot regions, the market share changes of originators, GCE certificated generics, and GCE uncertificated generics all had no significance (all *p*-values > 0.05).

**FIGURE 4:**
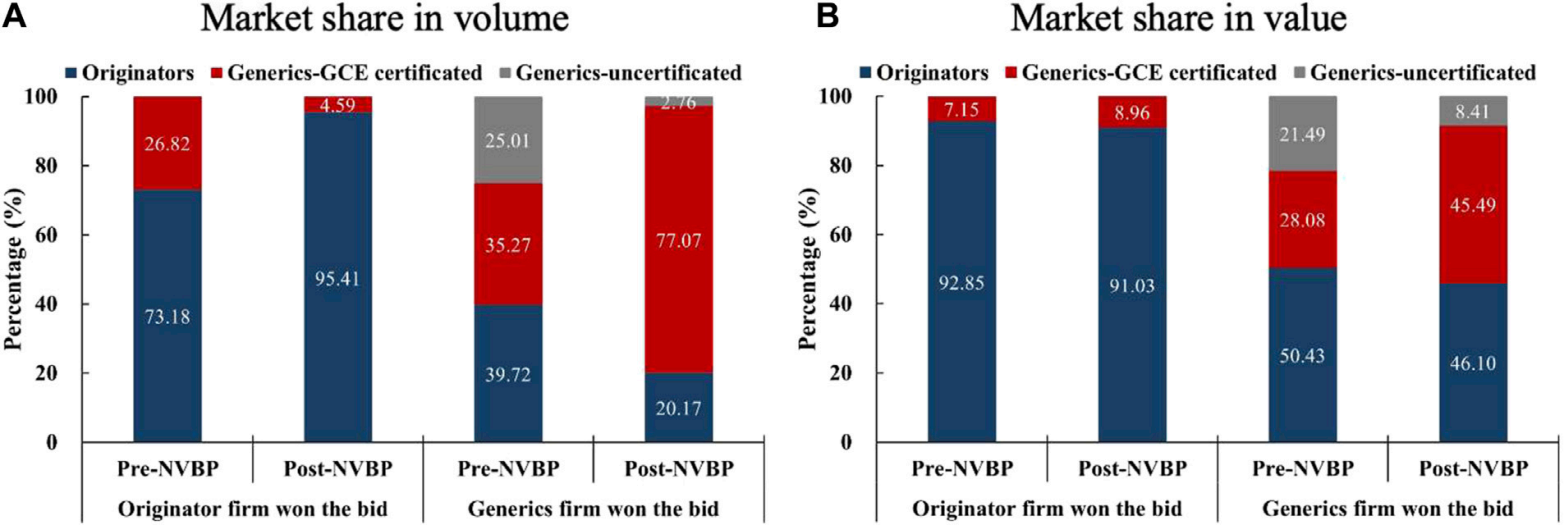
Changes in the market share (in volume and value) among three product groups (originators, GCE certificated generics, and GCE uncertificated generics) by bid-winner’s type in pilot regions. **(A)** Market share in volume, **(B)** market share in value. Note: GCE, generic consistency evaluation; NVBP, national volume-based procurement.

Twenty-five include drugs were further dichotomized by the characteristic of bid-winners: a) INNs that were won the bid by the originator pharmaceutical enterprise (*n* = 3), namely Gefitinib, Fosinopril, and Flurbiprofen; and b) INNs that were won by the generic pharmaceutical enterprise (*n* = 22). Figure 4 displayed the market share changes of the two groups in pilot regions. For the three INNs that won the bid by originators, the market share of GCE uncertificated generics had been 0, while originators increased from 73.18% to 95.41% in volume share after policy intervention, with value share generally unchanged (92.85% vs. 91.03%). For the twenty-two INNs that won the bid by generics, the above characteristic of “one upward and two downwards” was presented markedly, with the market share of GCE certificated generics reaching 77.07% (in volume) and 45.49% (in value) in the post-NVBP period.

### 4.3 Market concentration

#### 4.3.1 The change of market concentration

**FIGURE 5:**
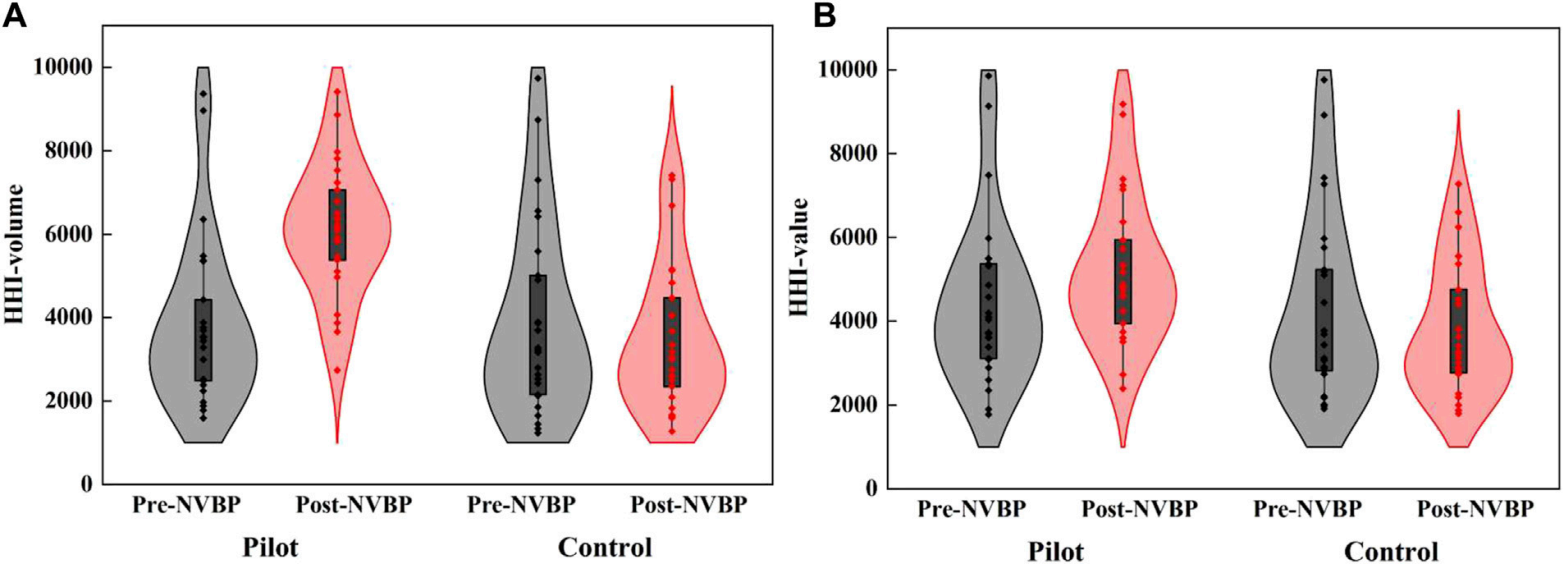
The distribution of HHI-volume and HHI-value in twenty-five included INNs before and after NVBP implementation. **(A)** HHI-volume, **(B)** HHI-value. Note: HHI, Herfindahl-Hirschman index; NVBP, national volume-based procurement.

In pilot regions, the average level of HHI among 25 INNs improved remarkedly after NVBP policy, with an increment of 2,266.23 (from 3,851.18 to 6,117.41) for HHI-volume and 722.17 (from 4,468.90 to 5,191.07) for HHI-value. In contrast, HHI in non-pilot regions slightly declined, with a decrease of 414.70 (from 3,901.81 to 3,487.11) for HHI-volume and 468.49 (from 4,273.10 to 3,804.61) for HHI-value. Specifically for each INN, only 3 exhibited a decrease in HHI-volume and 6 exhibited a decrease in HHI-value in pilot regions. Full results of market concentration changes for each INN are listed in Supplementary Appendix D. The violin plot in Figure 5 depicts the distribution of market concentration in 25 INNs before and after NVBP policy. It can be seen that, in pilot regions, the distribution range of both HHI-volume and HHI-value moved up after policy intervention, and the former was more obvious than the latter. Whereas, the distribution of HHI showed little change in non-pilot regions.

#### 4.3.2 Model estimation on market concentration

Model estimation results of the above Formula (2) showed that (Table 2), the effects of NVBP policy on both HHI-volume and HHI-value were significantly positive at the statistical level of 1%. According to columns (3) and (6), the HHI-volume and HHI-value significantly increased by 49.33% (e^0.401–1^ = 0.4933) and 21.05% (e^0.191–1^ = 0.2105), respectively. The corresponding parallel trend test results of all DID estimation models are detailed in Supplementary Appendix D.

**TABLE 2:** Model estimation on the impact of NVBP policy on market concentration.

	HHI-volume			HHI-value		
	(1)	(2)	(3)	(4)	(5)	(6)
*NVBP*	0.336***	0.398***	0.401***	0.131***	0.189***	0.191***
	(0.010)	(0.014)	(0.010)	(0.010)	(0.014)	(0.009)
*lngdp*	−0.498***	−0.138	−0.114	−0.312***	0.053	0.062
	(0.100)	(0.124)	(0.099)	(0.095)	(0.117)	(0.086)
*lnpop.*	−0.860***	−0.732***	−0.719***	−0.648***	−0.501***	−0.516***
	(0.156)	(0.161)	(0.110)	(0.151)	(0.156)	(0.102)
*lnbeds*	−0.535**	0.436	0.392**	−0.576***	0.382	0.357**
	(0.212)	(0.286)	(0.197)	(0.204)	(0.272)	(0.179)
*Constant*	22.861***	13.789***	13.597***	19.386***	10.175***	10.282***
	(1.660)	(2.555)	(2.036)	(1.615)	(2.423)	(1.777)
*Drug FE*	Y	Y	N	Y	Y	N
*Region FE*	Y	Y	N	Y	Y	N
*Time FE*	N	Y	Y	N	Y	Y
*Drug-Region FE*	N	N	Y	N	N	Y
*N*	12,044	12,044	12,043	12,044	12,044	12,043
*Adjusted R* ^*2*^	0.511	0.514	0.778	0.447	0.451	0.781

Note: *, **, and *** indicate significance at 10%, 5%, and 1% level. HHI, Herfindahl-Hirschman index; NVBP, national volume-based procurement; FE, fixed effect.

Subgroup analysis was conducted by considering the baseline market share level of bid-winners, as shown in Table 3. First of all, whether the baseline market share of the bid-winner was high, medium, or low, the impact of NVBP policy on both HHI-volume and HHI-value was significantly positive at the statistical level of 1%, which reflected the robustness of the benchmark estimation results. Secondly, a positive correlation between the baseline market share level of bid-winner and the improvement of market concentration was observed: the increment of HHI-volume in low, medium, and high baseline market share group was 46.37% (e^0.381–1^ = 0.4637), 49.48% (e^0.402–1^ = 0.4948), and 52.96% (e^0.425–1^ = 0.5296) respectively, and the increment of HHI-value was 18.29% (e^0.168–1^ = 0.1829), 20.32% (e^0.185–1^ = 0.2032), and 24.98% (e^0.223–1^ = 0.2498) respectively.

**TABLE 3:** Subgroup analysis on the impact of NVBP policy on market concentration by bid-winner’s baseline market share level.

	HHI-volume			HHI-value		
	Low	Medium	High	Low	Moderate	High
	(1)	(2)	(3)	(4)	(5)	(6)
*NVBP*	0.381***	0.402***	0.425***	0.168***	0.185***	0.223***
	(0.019)	(0.016)	(0.017)	(0.017)	(0.015)	(0.014)
*lngdp*	−0.686***	0.098	0.170	−0.132	0.005	0.290**
	(0.199)	(0.160)	(0.142)	(0.157)	(0.160)	(0.116)
*lnpop.*	−1.187***	−0.540***	−0.508***	−0.893***	−0.549***	−0.170
	(0.199)	(0.184)	(0.180)	(0.182)	(0.176)	(0.169)
*lnbeds*	−1.266***	1.365***	0.933***	−0.818**	1.113***	0.687**
	(0.360)	(0.322)	(0.337)	(0.329)	(0.312)	(0.282)
*Constant*	30.400***	5.750*	6.696**	20.153***	7.984**	3.829
	(4.075)	(3.363)	(2.845)	(3.273)	(3.267)	(2.441)
*N*	3,859	4,356	3,828	3,859	4,356	3,828
*Adjusted R* ^*2*^	0.797	0.745	0.752	0.790	0.732	0.797

Note: *, **, and *** indicate significance at 10%, 5%, and 1% level. Fixed effects were applied including *time* fixed effect and *drug-region* fixed effect. HHI, Herfindahl-Hirschman index; NVBP, national volume-based procurement.

We further conducted a subgroup analysis by considering the bid-winning enterprise’s characteristics. As shown in Table 4, whether was originator or generic won the bid, the impact of NVBP policy on market concentration was significantly positive at the 1% statistical level. In the group that originators won the bid, the increment of HHI-volume and HHI-value was 30.47% (e^0.266–1^ = 0.3047) and 25.61% (e^0.228–1^ = 0.2561), at a comparable level. While in the group that generics won the bid, the increment of HHI-volume (52.50%, e^0.422–1^ = 0.5250) is much greater than that of HHI-value (20.56%, e^0.187–1^ = 0.2056).

**TABLE 4:** Subgroup analysis on the impact of NVBP policy on market concentration by bid-winning enterprise’s characteristic.

	HHI-volume		HHI-value	
	Originators won the bid	Generics won the bid	Originators won the bid	Generics won the bid
	(1)	(2)	(3)	(4)
*NVBP*	0.266***	0.422***	0.228***	0.187***
	(0.023)	(0.011)	(0.021)	(0.010)
*lngdp*	0.755***	−0.237**	0.568***	−0.010
	(0.161)	(0.109)	(0.144)	(0.095)
*lnpop.*	0.975***	−0.955***	0.996***	−0.731***
	(0.209)	(0.121)	(0.211)	(0.112)
*lnbeds*	0.028	0.434**	−0.335	0.444**
	(0.416)	(0.214)	(0.382)	(0.196)
*Constant*	−7.130**	16.542***	−3.635	12.311***
	(3.316)	(2.238)	(3.030)	(1.973)
*N*	1,414	10,629	1,414	10,629
*Adjusted R* ^*2*^	0.480	0.766	0.523	0.764

Note: *, **, and *** indicate significance at 10%, 5%, and 1% level. Fixed effects were applied including *time* fixed effect and *drug-region* fixed effect. HHI, Herfindahl-Hirschman index; NVBP, national volume-based procurement.

## 5 Discussion

### 5.1 NVBP promoted the intensification of drug supply in hospital-end market

This study found that the number of products circulated in the hospital-end market decreased significantly after the NVBP policy, and the number of manufacturing enterprises reduced for some NVBP drugs. Under the existing regulation of “one drug with two specifications”^3^ in hospital drug procurement and use of the Chinese government, the implementation of unified bidding and joint procurement after NVBP policy would inevitably increase product overlap among hospitals and regions (gathering on bid-winning products), as well as decreasing circulated products number in the hospital-end market. In China, there are too many domestic pharmaceutical products, the approval number of domestic chemical products reached about 107,000 and most of which are “zombie approval numbers” (Ding, 2019). There is a report claiming that NVBP policy has improved the utilization rate of drug approval numbers and promoted the elimination of “zombie” numbers (Sina Finance, 2023), which supported the findings of the present study. However, the intensification of drug supply is a double-edged sword, and previous studies have reported hospital shortage for some low-priced, high clinical demand medicines in certain regions following the implementation of NVBP policy (Zhou et al., 2023; Wang et al., 2024). Long-term tracking and observation are required for identifying more accurate industry trends. In the future context of normalized and institutionalized NVBP, it is necessary to continuously monitor hospital drug supply and use of policy-covered and policy-related drugs, especially for products with very low bidding prices and bid-winning enterprises with production capacity risks (Li et al., 2022).

### 5.2 GCE combined with NVBP brought chances for domestic industry

NVBP policy enabled the practical full implementation of GCE criteria in China’s hospital-end market. In this study, it is easy to see that the market share of enterprises with GCE uncertificated generics has been maximally squeezed and compressed after NVBP intervention, regardless of the type of bid-winners. According to the State Council documents introduced in 2016 (General Office of the State Council, 2016) and 2021 (General Office of the State Council, 2021), it was emphasized that when purchasing drug INN with more than 3 enterprises gotten GCE certification, GCE uncertificated products were not allowed in principle. This means that generics can hardly circulate in the hospital-end market without GCE certification, which is conducive to creating a development concept of the pharmaceutical industry that emphasizes drug quality.

This study found that, after winning the bid in NVBP, pharmaceutical enterprises with lower baseline market share gained far more remarkable increment in market share, and their market benefit might be much higher than those with a higher baseline market share. We believe this is an important pathway for small-scale enterprises to survive and improve their market position in the context of NVBP policy. Chen et al. (2022) found a “basin effect” in the relationship between the enterprise scale ranking (by the main business income) of bid-winners and their winning number of products under NVBP policy, that is, small-scale (below 400th rank) enterprises accounted for 20%–40% of the winning products, lower than that of the top 100 enterprises (51%–71%), and much higher than that of the medium-scale (101th to 400th rank) enterprises (0%–11%). Moreover, from the first to fourth round pilot, the proportion of winning products by below 400th rank enterprises increased, while that of the top 100 enterprises decreased. It can be seen that small-scale enterprises are actively seizing the opportunity of NVBP, striving to gain hospital market share increment through bid-winning and price reduction.

However, we also found the mismatch between the increment in value share and volume share of bid-winning enterprises, that is, a significant price cut in NVBP leads to a prominent increase in sales volume and only a small increase in sales value, which showed enterprise’s original intension of cutting prices in exchange for sales. The NVBP mechanism changed the drug commercial model and significantly reduced enterprise costs such as academic promotion expenses and period expenses (Wang et al., 2019). Whereas, low procurement prices and large procurement quantity posed higher requirements for the enterprise’s cost control and supply capacity, challenging their profitability and sustainable development. Hua et al. (2022) found in their study on corporate profitability that the formal implementation of NVBP negatively impacted the net profit of pharmaceutical enterprises, and pointed out that generic pharmaceutical enterprises need to strengthen R&D capacity as soon as possible to hedge against these “losses.” The practice in Sweden indicated that the procurement mechanism pursuing low prices can improve the market share of low-priced products and save drug expenditures in the short term, but the long-term effect was negligible (Bergman et al., 2017). Therefore, in the promoting process of normalized and institutionalized NVBP, it is necessary to gain a balance between low bid prices and sustainable industrial development, in order to achieve an organic combination of moderate government and market mechanisms.

### 5.3 The potential “exit” strategy of original brand-name enterprises

This study found that, after the NVBP policy, original brand-name products that failed to win the bid could still maintain an average value share of about 45% in the hospital-end market, and this figure even reached over 70% for some INNs. With the addition of strong competitive advantage of original drugs in the retail market, it is indicated that not winning the bid in NVBP might not bring significant losses to the original drug enterprises on the whole. On the contrary, we found that original drug enterprises mainly gained market improvement in volume share after winning the bid in NVBP, while the value share did not. In this situation, original pharmaceutical enterprises may prefer an “exit” strategy—exit from the hospital-end market. In the case of Acarbose during the second round NVBP (Jiemian News, 2022), the original brand enterprise (Bayer) won the bid with a far lower price than corresponding generic enterprise, while the significant increase in its market share cannot offset the income loss caused by the price reduction in NVBP. Bayer even suffered a situation of “selling more, losing more” due to excessive growth of product demand, subsequently, it gave up the renewal contract of NVBP in multiple provinces. Some scholars have pointed out that NVBP is a “life and death” issue for domestic pharmaceutical enterprises and more of a strategic issue for foreign-invested enterprises, thus many original brand enterprises will shift their layout from hospital-end to the retail-end drug market in the future (Xu, 2019; Gao, 2021). In the context of immature prescription outflow mechanism in China’s public medical institutions, the lack of original drugs in the hospital-end market may bring some inconvenience, for example, patient dissatisfaction caused by the unavailability of original drugs within hospitals, unnecessary travel caused by obtaining original drugs from retail pharmacies, and increased drug cost caused by higher drug prices at retail pharmacies compared to hospitals.

### 5.4 NVBP mode improved industry concentration

In the process of China’s pharmaceutical supply-side structural reform, it is a great challenge to alter the industry situation of “many, scattered, and small,” and improve the industry concentration. This study indicated that the implementation of NVBP had significantly improved market concentration in the hospital-end drug market, moreover, it will raise greater concentration increment when enterprises with higher baseline market share won the bid. This means, guiding more large-scale enterprises to participate in NVBP policy and win the bid will promote industrial concentration to a greater extent. Globally, developed countries such as the United States and the United Kingdom which have early implemented centralized drug procurement mechanisms, exhibit high market concentration and rapid industrial development (She and Wen, 2016; Shi et al., 2022). In contrast, developing countries with centralized procurement mechanisms like India, also demonstrated higher market concentration than China (Cui et al., 2022; Mordor Intelligence, 2023). The centralized procurement mechanism serve as an important driving force for the intensive development of national pharmaceutical industries. Considering its national conditions, China has established a drug centralized procurement mechanism underlying the GCE, which has significantly contributed to the consolidation of pharmaceutical industry in the short term. This may offer practical insights for other developing countries facing similar pharmaceutical industry challenges of high industry decentralization and low industry quality.

### 5.5 Limitations and prospects

Firstly, it is well known that the NVBP mode had brought substantial increase in product sales of bid-winning enterprises both in volume and value (Chen et al., 2020; Wang et al., 2022). When attempting to explore the impact of NVBP policy on specific pharmaceutical enterprises, the savings in channel expenses and marginal production costs brought about by the adjustment of procurement mechanisms should be considered (Tao, 2020; Luo et al., 2022), that is, the net profit and operation performance of a specific enterprise under NVBP implementation are of more reference value. However, in this study, due to the lack of cost and profit data for specific enterprises targeting specific drugs, we cannot accurately disclose the actual impact of NVBP implementation on the operation performance of a specific enterprise. Existing literature provided inconsistent clues. For example, some scholars (Li and Shen, 2020; Wang, 2020; Xu, 2022) observed a downward trend in marketing expenses and an increasing trend in gross profit for Huahai Pharmaceuticals after winning the NVBP bid, believing a positive policy impact on enterprise performance. While Hua et al. (2022)’s empirical research on 65 listed companies shows that the NVBP policy has a significant negative impact on the net profit of pharmaceutical companies. Therefore, future studies are needed to strengthen the integration analysis of macro (industry-level) and micro (enterprise-level) data, and to improve the accuracy of impact effect estimation.

Secondly, the observation of large-scale industrial restructuring generally requires a longer follow-up time (Yang et al., 2023). While this study only conducted a short-term (23 months) and small-scale (25 drug INNs) observation, the current results might be incomplete in revealing the overall trend of China’s pharmaceutical industry under the NVBP policy. In addition, the NVBP system has been dynamically adjusted since its launch in 2018 (Chang, 2021; Xue et al., 2022), including the scope of drugs to be procured, the scope of procurement institutions, bid evaluation rules, and the assignment of drug suppliers, etc. For example, the single-supplier mode was applied in the first round NVBP pilot that this study focused on, and it was subsequently adjusted to a multi-suppliers approach in the latter pilot rounds. Thus, the adjustment of procurement rules among NVBP pilot rounds might contribute to the non-universality of the present research findings to some extent. Volume-based procurement is rapidly advancing in the Chinese mainland, at the national level, seven rounds of NVBP pilots were implemented as of September 2022, with 294 drugs successfully procured (National Healthcare Security Administration, 2023); at the local level, 23 provincial purchasing alliances were organized as of February 2022, with over 500 drugs procured (Weng et al., 2022). Therefore, under the rapidly changing policy situation, follow-up studies are needed to expand the scope of policy observation and evaluation in a timely and long-term manner, to reflect the changes and trends of the Chinese pharmaceutical industry more accurately.

## 6 Conclusion

In China, the implementation of NVBP policy brought positive signals for pharmaceutical industry optimization that promoted the intensification of drug circulation and supply in the hospital-end market, which might mainly benefit from the withdrawal of “zombie” numbers and GCE uncertificated generic products. Meanwhile, it is necessary to continuously monitor hospital drug supply and be vigilant against the risk of drug shortage might cause by excessive intensification.

The combination of NVBP and GCE might accelerate the pace of low-end production capacity of the Chinese pharmaceutical industry exiting the hospital-end market. For Chinese pharmaceutical enterprises, seeking NVBP winning is a survival opportunity in the short term, while investing in drug R&D to expand profit sources beyond generic drugs is an inevitable trend in the long term.

NVBP policy has significantly improved market concentration in the Chinese hospital-end market, which is conducive to pharmaceutical industrial restructuring. On the one hand, more benefits (greater market concentration increment, low drug supply risk, etc.) could be brought when enterprises with higher baseline market share won the bid. On the other hand, overseas original brand-name enterprises might be inclined to abandon the Chinese hospital-end market under the NVBP policy. Contradictions still exist, and the NVBP policy needs to further seek the balance between drug price and stable hospital drug supply, as well as sustainable development of the Chinese pharmaceutical industry.

## Data Availability

The original contributions presented in the study are included in the article/Supplementary material, further inquiries can be directed to the corresponding authors.

## Footnotes

1 Based on the definition of National Bureau of Statistics, the large-scale manufacturing enterprise size refers to industrial legal entity with an annual main business income of ≥20 million Chinese yuan.

2 Products reached GCE quality standard include four categories: (a) GCE certificated generic drug, (b) original brand-name drug, (c) reference product, and (d) product that approved for marketing according to China’s new regulation of chemical medicine registration and classification.

3 The “Prescription Administrative Regulation” of Chinese government stipulated that the injectable forms and oral forms of drugs with the same generic name shall not exceed two in the hospital.
