# Supplementary material for: The impact of Chinese volume-based procurement on pharmaceutical market concentration
Source: Front Pharmacol. 2024 Jun 4;15:1386533. doi: 10.3389/fphar.2024.1386533 (PMC11183305; doi:10.3389/fphar.2024.1386533)
Supplement: Supplementary file 1 [file Table1.DOCX]

Supplementary Material

# Appendix A. Outline of included drug samples

## **Table A.** List of 25 drugs procured in the first round NVBP pilot.

| No. | Name of drug ingredient | ATC code | DDD value | Drug category | Bid-winning enterprise |
| --- | --- | --- | --- | --- | --- |
| 1 | Amlodipine | C08CA01 | 5mg | Antihypertensive drug | Jingxin Pharmaceuticals |
| 2 | Losartan | C09CA01 | 50mg | Antihypertensive drug | Huahai Pharmaceuticals |
| 3 | Irbesartan | C09CA04 | 150mg | Antihypertensive drug | Huahai Pharmaceuticals |
| 4 | Irbesartan and Hydrochlorothiazide | C09DA04 | 162.5mg | Antihypertensive drug | Huahai Pharmaceuticals |
| 5 | Fosinopril | C09AA09 | 15mg | Antihypertensive drug | Squibb Pharmaceuticals* |
| 6 | Lisinopril | C09AA03 | 10mg | Antihypertensive drug | Huahai Pharmaceuticals |
| 7 | Enalapril | C09AA02 | 10mg | Antihypertensive drug | Yangtze River Pharmaceuticals |
| 8 | Atorvastatin | C10AA05 | 20mg | Lipid modifying agents | Jialin Pharmaceuticals |
| 9 | Rosuvastatin | C10AA07 | 10mg | Lipid modifying agents | Jingxin Pharmaceuticals |
| 10 | Levetiracetam | N03AX14 | 1500mg | Antiepileptics | Jingxin Pharmaceuticals |
| 11 | Olanzapine | N05AH03 | 10mg | Psycholeptics | Hansoh Pharmaceuticals |
| 12 | Risperidone | N05AX08 | 5mg | Psycholeptics | Huahai Pharmaceuticals |
| 13 | Dexmedetomidine | N05CM18 | 1mg | Psycholeptics | Yangtze River Pharmaceuticals |
| 14 | Escitalopram | N06AB10 | 10mg | Psychoanaleptics | Sichuan Kelun Pharmaceuticals |
| 15 | Paroxetine | N06AB05 | 20mg | Psychoanaleptics | Huahai Pharmaceuticals |
| 16 | Gefitinib | L01EB01 | 250mg | Antineoplastic agents | AstraZeneca* |
| 17 | Imatinib | L01XE01 | 500mg | Antineoplastic agents | Hansoh Pharmaceuticals |
| 18 | Pemetrexed | L01BA04 | 44.05mg | Antineoplastic agents | Huiyu Pharmaceuticals |
| 19 | Cefuroxime | J01DC02 | 500mg | Antibacterials for systemic use | Brilliant Pharmaceuticals |
| 20 | Entecavir | J05AF10 | 0.5mg | Antivirals for systemic use | Zhengda Tianqing Pharmaceuticals |
| 21 | Tenofovir Disoproxil | J05AF07 | 245mg | Antivirals for systemic use | Brilliant Pharmaceuticals |
| 22 | Montmorillonite | A07BC05 | 9000mg | Antidiarrheals, intestinal  antiinflammatory/antiinfective agents | Simcere Pharmaceuticals |
| 23 | Clopidogrel | B01AC04 | 75mg | Antithrombotic agents | Salubris Pharmaceuticals |
| 24 | Flurbiprofen | M01AE09 | 50mg | Antiinflammatory and antirheumatic products | Tide Pharmaceuticals* |
| 25 | Montelukast | R03DC03 | 10mg | Drugs for obstructive airway diseases | Anbisheng Pharmaceuticals |

* indicate original brand-name pharmaceutical enterprise. ATC, anatomical therapeutic chemical; DDD, defined daily dosage.

DDDs represents the consumed volume of a certain drug (or a group of drugs). For each drug, DDDs was calculated as

$$\text{DDD}_{\text{s}}\text{=}\sum_{\text{i=1}}^{\text{n}} \text{(}{\frac{\text{U}_{\text{i}}\text{P}_{\text{i}}}{\text{DDD}}}_{\text{i}}\text{×}\text{N}_{\text{i}}\text{)}$$

where *DDD_i_* refers to the DDD value of drug product *i*, *U_i_* refers to the unit ingredient of product *i* (i.e. the content of each pill or capsule), *P_i_* refers to the packing specification product *i* (e.g. the number of tablets contained in each box), and *N_i_* refers to the accumulated quantity of product *i* consumed in terms of package units.

# Appendix B. The procedure of determining the control group

**Step one: Stratification of observation regions**

This study first stratified the observation cities/provinces by geographical regions, mainly considering the following three aspects. Firstly, due to regional cultural differences in China, there are differences in the drug usage habits and medication structures of Chinese residents among geographical regions. Secondly, the distribution of China’s pharmaceutical industry is characterized by regional imbalance. For example, in the Chinese mainland, the vast majority of chemical pharmaceutical enterprises are concentrated in economically developed areas along the eastern coast, while the traditional Chinese medicine industry is mainly concentrated in the western and central regions (Wan and Zhang, 2015; Wang et al., 2017), resulting in regional differences in drug sales and drug market patterns. Thirdly, the inherent regional disparity in economic level and health resources distribution brings differences in medical care and medication among regions (Yan and Yan, 2019). In this study, the eleven included pilot cities are distributed in Eastern China (Xiamen, Shanghai), North China (Tianjin, Beijing), Central and Southern China^[[1]](#footnote-0)^ (Guangzhou, Shenzhen), Northeast China (Dalian, Shenyang), Southwest China (Chengdu, Chongqing), and Northwest China (Xi’an), respectively. Therefore, select control regions from these six major geographical regions.

**Step two: Selection of matching variables**

In this study, the geographical region was used as the observation unit for matching, and the matching variables should include factors closely related to drug utilization and policy implementation in the region. Referring to the practice in existing literature (Li et al., 2021; Tang et al., 2016) and preliminary published works of our team (Lu et al., 2022; Wang et al., 2022), the present study selected matching variables from four dimensions: population size, economic level, medical resources, and medical services. Finally, nine indicators were included, that is, year-end permanent population (10,000 people), per capita gross domestic product (GDP) (100 million Chinese yuan), number of health institutions (per 1000 population), number of hospital beds (per 1000 population), number of skilled health workers (per 1000 population), number of licensed (assistant) doctors (per 1000 population), per capita health expenditures (Chinese yuan), annual average clinical visits, and annual hospitalization rate (%).

**Step three: Stratified matching and the result of control group selection**

In this study, the TOPSIS (Technique for Order of Preference by Similarity to Ideal Solution) method was applied to match control regions, mainly from the following three considerations. Firstly, the overall sample size of the pilot group was small, with only 11 cities. Secondly, under the requirement of stratified matching by geographical region, each geographical region group only had 1-2 pilot group samples. Thirdly, the original matching degree between the pilot and the non-pilot regions was not high^[[2]](#footnote-1)^, and it was difficult to complete the matching using methods such as propensity score matching (PSM) while retaining all the pilot group samples. Therefore, the more operational unweighted TOPSIS method was applied. Within the corresponding geographical region, the non-pilot province that had the closest TOPSIS score to the pilot city was selected into the control group.

The results of TOPSIS scores are presented in **Table A** below. Eleven non-pilot provinces were selected as control regions, i.e. Jiangsu, Zhejiang, Inner Mongolia, Shanxi, Hubei, Hunan, Jilin, Heilongjiang, Guizhou, Tibet, and Qinghai. In addition, it should be mentioned that, in the retrospective CDSIP data applied in this study, the data statistics of non-pilot regions (except for eleven pilot cities) were carried out by the province. We were unable to obtain control group samples with city units, which may, to some extent, result in poor original comparability between pilot and control regions.

## **Table A.** Results of (unweighted) TOPSIS.

| Regions | | Per capita GDP (100 million CNY) | Population size (10,000) | Number of health institutions (per 1000 population) | Number of hospital beds (per 1000 population) | Number of skilled health workers (per 1000 population) | Number of licensed (assistant) doctors (per 1000 population) | Per capita health expenditure (CNY) | Annual average clinical visits | Annual hospitalization rate (%) | TOPSIS  Score |
| --- | --- | --- | --- | --- | --- | --- | --- | --- | --- | --- | --- |
| Eastern China | Xiamen* | 142739.00 | 429.00 | 0.49 | 4.38 | 8.61 | 3.54 | 4276.70 | 6.27 | 15.30 | 0.1665 |
|  | Shanghai* | 157279.00 | 2428.00 | 0.23 | 6.03 | 8.40 | 3.10 | 10430.53 | 11.35 | 18.70 | 0.3228 |
|  | Jiangsu⟊ | 123607.00 | 8070.00 | 0.43 | 6.39 | 7.80 | 3.20 | 5525.61 | 7.65 | 18.90 | 0.2546 |
|  | Zhejiang⟊ | 107624.00 | 5850.00 | 0.58 | 5.99 | 8.90 | 3.50 | 5881.25 | 11.65 | 18.90 | 0.2856 |
| North China | Tianjin* | 90371.00 | 1562.00 | 0.38 | 4.37 | 7.00 | 3.00 | 6233.15 | 7.87 | 10.90 | 0.1338 |
|  | Beijing* | 164220.00 | 2154.00 | 0.48 | 5.93 | 12.60 | 4.90 | 13766.77 | 11.56 | 17.90 | 0.6110 |
|  | Inner Mongolia ⟊ | 67852.00 | 2540.00 | 0.97 | 6.34 | 7.70 | 3.10 | 4604.72 | 4.21 | 14.30 | 0.1526 |
|  | Shanxi⟊ | 45724.00 | 3729.00 | 1.13 | 5.86 | 6.90 | 2.80 | 3465.29 | 3.53 | 13.40 | 0.1550 |
| Central and Southern China | Guangzhou* | 131400.00 | 1831.21 | 0.28 | 5.47 | 9.18 | 3.20 | 5317.60 | 7.74 | 15.80 | 0.1797 |
|  | Shenzhen* | 159883.00 | 1710.40 | 0.29 | 2.81 | 6.01 | 2.36 | 5317.60 | 7.74 | 15.80 | 0.1868 |
|  | Hubei⟊ | 77387.00 | 5927.00 | 0.60 | 6.80 | 7.00 | 2.60 | 4354.57 | 5.97 | 23.10 | 0.1897 |
|  | Hunan⟊ | 57540.00 | 6918.00 | 0.83 | 7.32 | 7.30 | 2.80 | 4006.25 | 4.06 | 23.40 | 0.2056 |
| Northeast China | Dalian* | 99996.00 | 598.70 | 0.69 | 8.22 | 9.58 | 3.85 | 4160.65 | 4.59 | 16.30 | 0.1639 |
|  | Shenyang* | 77777.00 | 755.40 | 0.69 | 9.52 | 10.82 | 4.21 | 4160.65 | 4.59 | 16.30 | 0.1801 |
|  | Jilin⟊ | 43475.00 | 2691.00 | 0.82 | 6.33 | 7.00 | 2.90 | 4356.76 | 4.10 | 15.00 | 0.1258 |
|  | Heilongjiang⟊ | 36183.00 | 3751.00 | 0.54 | 7.00 | 6.30 | 2.50 | 4027.08 | 3.00 | 16.10 | 0.1180 |
| Southwest China | Chengdu* | 103386.00 | 1658.10 | 0.73 | 8.98 | 11.16 | 4.13 | 4424.37 | 6.69 | 23.70 | 0.2146 |
|  | Chongqing* | 75828.00 | 3124.00 | 0.67 | 7.42 | 7.20 | 2.70 | 4530.36 | 5.62 | 24.10 | 0.1612 |
|  | Guizhou⟊ | 46433.00 | 3623.00 | 0.79 | 7.31 | 7.40 | 2.50 | 3838.96 | 4.85 | 23.70 | 0.1653 |
|  | Tibet⟊ | 48902.00 | 351.00 | 1.98 | 4.87 | 6.00 | 2.70 | 5395.01 | 4.66 | 8.70 | 0.4261 |
| Northwest China | Xi'an* | 92256.00 | 1020.35 | 0.69 | 7.11 | 11.00 | 10.65 | 4706.80 | 5.39 | 21.10 | 0.4569 |
|  | Qinghai⟊ | 48981.00 | 608.00 | 1.07 | 6.82 | 7.80 | 2.90 | 5843.23 | 4.38 | 17.40 | 0.4978 |

* indicate pilot regions; ⟊ indicate control regions. GDP, gross domestic product; CNY, Chinese yuan.

**Step four: Comparability between pilot and control regions**

Firstly, analysis of variance was used to compare TOPSIS scores between the pilot and control groups. The average score of the pilot regions (0.25±0.151) was slightly higher than that of the control regions (0.23±0.125), but the difference was not statistically significant (*F*=0.096, *p*=0.760). The result showed that the macro-environmental factors (population, economy, medical resources, health services) of the pilot and the control group were generally balanced.

Secondly, paired *t*-test was used to compare the differences in drug market volume and value between the pilot and control group in the baseline (pre-NVBP) period. As shown in **Table B** below, no statistically significant difference was observed in baseline market capacity (volume and value) between the two groups (all *p*-values>0.05), whether using drug ingredient or enterprise as statistical units. The results indicated that the two groups are relatively comparable in the baseline period.

## **Table B.** Comparison of market capacity between pilot and control group in the baseline period.

|  | *n* | Pilot group | Control group | Diff | *t* | *p*-value |
| --- | --- | --- | --- | --- | --- | --- |
| *Ingredient level* |  |  |  |  |  |  |
| Volume (million DDD) | 25 | 127.00 (36.70) | 134.00 (39.40) | -6.63 (11.70) | -0.565 | 0.577 |
| Value (million CNY) | 25 | 837.00 (205.00) | 784.00 (169.00) | 52.90 (57.90) | 0.912 | 0.371 |
| *Enterprise level* |  |  |  |  |  |  |
| Volume (million DDD) | 206 | 15.50 (3.60) | 16.30 (2.91) | -0.80 (1.70) | -0.472 | 0.637 |
| Value (million CNY) | 206 | 102.00 (24.50) | 95.10 (20.10) | 6.42 (8.28) | 0.775 | 0.439 |

DDD, defined daily dosage; CNY, Chinese yuan

**References**

Li, Z., Liu, C., Zuo, K., Liu, J., Tang, Y., 2021. Effects of volume-price contracts on pharmaceutical prices: a retrospective comparative study of public hospitals in Hubei of China. Frontiers in Pharmacology, 12:741671. http://doi.org/10.3389/fphar.2021.741671.

Lu, J., Long, H., Shen, Y., Wang, J., Geng, X., Yang, Y., Mao, Z., Li, J., 2022. The change of drug utilization in China's public healthcare institutions under the “4+7” centralized drug procurement policy: evidence from a natural experiment in China. Frontiers in Pharmacology, 13:923209. http://doi.org/ 10.3389/fphar.2022.923209.

Tang, Y., Liu, C., Zhang, X., 2016. Public reporting as a prescriptions quality improvement measure in primary care settings in China: variations in effects associated with diagnoses. Scientific Reports, 6(01):39361. http://doi.org/ 10.1038/srep39361.

Wan, Y., Zhang, H., 2015. Variance analysis on the regional differences about the industrial competitiveness of Chinese medicine. Economic Geography, 35(11):138-143,151. http://doi.org/10.15957/j.cnki.jjdl.2015.11.019.

Wang, H., Zhang, X., Zhao, Y., Shi, T., Yang, G., 2017. Analysis on regional distribution characteristics and changing trend of Chinese patent medicine industry. China Journal of Chinese Materia Medica, 42(14):2814-2819. http://doi.org/10.19540/j.cnki.cjcmm.20170609.001.

Wang, J., Yang, Y., Xu, L., Shen, Y., Wen, X., Mao, L., Wang, Q., Cui, D., Mao, Z., 2022. Impact of ‘4+7’ volume-based drug procurement on the use of policy-related original and generic drugs: a natural experimental study in China. BMJ Open, 12(03):e54346. http://doi.org/ 10.1136/bmjopen-2021-054346.

Yan, Y., Yan, Y., 2019. A study on efficiency of health resources allocation in China. Modern Hospital Management, 17(05):6-11. http://doi.org/10.3969/j.issn.1672-4232.2019.05.002.

# Appendix C. Full results of market share analysis

## **Table A.** Descriptive changes in market share of bid-winning enterprises before and after NVBP policy in pilot and control regions.

| Name of drug  ingredient | | Pilot regions | | | | | | |  | Control regions | | | | | | |
| --- | --- | --- | --- | --- | --- | --- | --- | --- | --- | --- | --- | --- | --- | --- | --- | --- |
|  |  | Volume share | | |  | Value share | | |  | Volume share | | |  | Value share | | |
|  |  | Pre-NVBP | Post-NVBP | △ |  | Pre-NVBP | Post-NVBP | △ |  | Pre-NVBP | Post-NVBP | △ |  | Pre-NVBP | Post-NVBP | △ |
| 1 | Amlodipine | 0.16 | 66.34 | 66.19 |  | 0.02 | 9.47 | 9.45 |  | 3.52 | 4.33 | 0.81 |  | 0.54 | 3.15 | 2.61 |
| 2 | Losartan | 14.51 | 77.73 | 63.23 |  | 10.64 | 47.15 | 36.51 |  | 25.34 | 18.91 | -6.44 |  | 20.78 | 30.36 | 9.58 |
| 3 | Irbesartan | 1.68 | 76.70 | 75.03 |  | 0.67 | 30.41 | 29.75 |  | 4.33 | 3.07 | -1.27 |  | 2.53 | 7.98 | 5.45 |
| 4 | Irbesartan and  Hydrochlorothiazide | 16.63 | 81.64 | 65.01 |  | 10.20 | 62.57 | 52.37 |  | 30.38 | 11.08 | -19.30 |  | 17.06 | 27.53 | 10.46 |
| 5 | Fosinopril | 100.00 | 100.00 | 0.00 |  | 100.00 | 100.00 | 0.00 |  | 100.00 | 100.00 | 0.00 |  | 100.00 | 100.00 | 0.00 |
| 6 | Lisinopril | 4.57 | 85.89 | 81.32 |  | 8.45 | 49.20 | 40.75 |  | 40.38 | 20.13 | -20.25 |  | 38.88 | 28.14 | -10.74 |
| 7 | Enalapril | 42.25 | 89.15 | 46.90 |  | 50.52 | 85.72 | 35.20 |  | 38.00 | 44.99 | 7.00 |  | 50.48 | 61.95 | 11.47 |
| 8 | Atorvastatin | 17.98 | 68.19 | 50.21 |  | 15.14 | 25.92 | 10.78 |  | 21.51 | 19.37 | -2.14 |  | 18.33 | 19.26 | 0.92 |
| 9 | Rosuvastatin | 64.89 | 91.88 | 27.00 |  | 65.98 | 79.46 | 13.48 |  | 56.36 | 60.87 | 4.51 |  | 61.66 | 69.31 | 7.65 |
| 10 | Levetiracetam | 1.92 | 32.44 | 30.52 |  | 1.45 | 24.57 | 23.11 |  | 2.03 | 6.11 | 4.08 |  | 1.63 | 15.71 | 14.08 |
| 11 | Olanzapine | 56.61 | 75.40 | 18.79 |  | 55.21 | 66.86 | 11.65 |  | 71.73 | 63.65 | -8.08 |  | 68.80 | 65.50 | -3.30 |
| 12 | Risperidone | 12.69 | 54.55 | 41.86 |  | 6.14 | 10.66 | 4.52 |  | 15.20 | 14.18 | -1.02 |  | 8.85 | 11.14 | 2.30 |
| 13 | Dexmedetomidine | 1.57 | 83.01 | 81.44 |  | 0.65 | 83.48 | 82.83 |  | 1.40 | 14.24 | 12.85 |  | 1.30 | 40.59 | 39.29 |
| 14 | Escitalopram | 12.13 | 55.47 | 43.33 |  | 7.93 | 34.76 | 26.83 |  | 24.89 | 23.83 | -1.07 |  | 19.07 | 25.56 | 6.49 |
| 15 | Paroxetine | 51.47 | 78.97 | 27.50 |  | 41.80 | 53.54 | 11.73 |  | 55.45 | 50.24 | -5.21 |  | 49.44 | 51.26 | 1.82 |
| 16 | Gefitinib | 76.09 | 83.50 | 7.41 |  | 85.26 | 83.47 | -1.78 |  | 76.72 | 41.79 | -34.93 |  | 83.69 | 70.81 | -12.88 |
| 17 | Imatinib | 79.82 | 81.47 | 1.65 |  | 29.23 | 27.89 | -1.34 |  | 76.20 | 74.97 | -1.23 |  | 26.09 | 27.50 | 1.41 |
| 18 | Pemetrexed | 41.29 | 61.92 | 20.63 |  | 36.48 | 54.04 | 17.56 |  | 65.49 | 64.30 | -1.20 |  | 69.70 | 62.92 | -6.78 |
| 19 | Cefuroxime | 8.60 | 77.45 | 68.84 |  | 4.40 | 46.15 | 41.75 |  | 3.25 | 5.83 | 2.57 |  | 1.60 | 10.95 | 9.35 |
| 20 | Entecavir | 71.56 | 97.48 | 25.91 |  | 84.46 | 93.73 | 9.27 |  | 65.11 | 60.98 | -4.13 |  | 72.30 | 72.97 | 0.67 |
| 21 | Tenofovir Disoproxil | 25.74 | 78.54 | 52.79 |  | 24.97 | 42.27 | 17.30 |  | 26.72 | 62.85 | 36.13 |  | 25.42 | 57.43 | 32.01 |
| 22 | Montmorillonite | 6.81 | 73.47 | 66.66 |  | 8.70 | 71.69 | 62.99 |  | 17.52 | 27.73 | 10.21 |  | 13.88 | 37.73 | 23.84 |
| 23 | Clopidogrel | 36.52 | 75.43 | 38.91 |  | 31.13 | 48.61 | 17.48 |  | 37.74 | 34.05 | -3.69 |  | 31.30 | 35.82 | 4.52 |
| 24 | Flurbiprofen | 96.73 | 93.92 | -2.81 |  | 99.27 | 95.74 | -3.53 |  | 98.66 | 84.03 | -14.63 |  | 98.80 | 86.46 | -12.34 |
| 25 | Montelukast | 0.00 | 51.48 | 51.48 |  | 0.00 | 29.53 | 29.53 |  | 0.00 | 0.28 | 0.28 |  | 0.00 | 13.22 | 13.22 |
|  | Total | 21.51 | 75.19 | 53.67 |  | 29.91 | 48.71 | 18.79 |  | 22.69 | 21.75 | -0.94 |  | 34.22 | 38.30 | 4.08 |

NVBP, national volume-based procurement; △ refers to the difference value between post- and pre-NVBP periods.

## **Table B.** Descriptive changes in market share structure among three categories (GCE uncertificated generics, GCE certificated generics, and originators) before and after NVBP policy in pilot and control regions.

|  | Name of drug  ingredient | Categories | Pilot Group | | | | | | |  | Control Group | | | | | | |
| --- | --- | --- | --- | --- | --- | --- | --- | --- | --- | --- | --- | --- | --- | --- | --- | --- | --- |
|  |  |  | Market share in volume | | |  | Market share in value | | |  | Market share in volume | | |  | Market share in value | | |
|  |  |  | Pre-  NVBP | Post-  NVBP | △ |  | Pre-  NVBP | Post-  NVBP | △ |  | Pre-  NVBP | Post-  NVBP | △ |  | Pre-  NVBP | Post-  NVBP | △ |
| 1 | Amlodipine | GCE uncertificated generics | 17.73 | 2.06 | -15.67 |  | 8.49 | 1.84 | -6.65 |  | 47.95 | 41.73 | -6.22 |  | 29.17 | 21.49 | -7.68 |
|  |  | GCE certificated generics | 39.23 | 74.41 | 35.18 |  | 20.00 | 19.09 | -0.91 |  | 31.23 | 36.02 | 4.78 |  | 21.36 | 30.63 | 9.27 |
|  |  | Originators | 43.04 | 23.54 | -19.51 |  | 71.51 | 79.07 | 7.56 |  | 20.81 | 22.25 | 1.44 |  | 49.47 | 47.88 | -1.59 |
| 2 | Losartan | GCE uncertificated generics | 43.86 | 3.69 | -40.17 |  | 42.59 | 8.46 | -34.13 |  | 53.74 | 51.68 | -2.06 |  | 48.13 | 45.08 | -3.06 |
|  |  | GCE certificated generics | 14.51 | 77.73 | 63.23 |  | 10.64 | 47.15 | 36.51 |  | 25.34 | 18.91 | -6.44 |  | 20.78 | 16.62 | -4.16 |
|  |  | Originators | 41.63 | 18.57 | -23.06 |  | 46.77 | 44.39 | -2.38 |  | 20.92 | 29.42 | 8.50 |  | 31.09 | 38.30 | 7.21 |
| 3 | Irbesartan | GCE uncertificated generics | 57.13 | 3.88 | -53.25 |  | 47.34 | 8.38 | -38.97 |  | 42.81 | 38.68 | -4.13 |  | 42.10 | 38.77 | -3.33 |
|  |  | GCE certificated generics | 8.80 | 77.39 | 68.58 |  | 3.57 | 31.13 | 27.57 |  | 35.68 | 37.80 | 2.12 |  | 19.44 | 19.53 | 0.09 |
|  |  | Originators | 34.06 | 18.73 | -15.34 |  | 49.09 | 60.49 | 11.40 |  | 21.50 | 23.52 | 2.02 |  | 38.46 | 41.70 | 3.24 |
| 4 | Irbesartan and  Hydrochlorothiazide | GCE uncertificated generics | 46.48 | 4.06 | -42.42 |  | 46.30 | 5.38 | -40.92 |  | 26.35 | 34.27 | 7.92 |  | 25.55 | 27.33 | 1.79 |
|  |  | GCE certificated generics | 33.69 | 85.62 | 51.93 |  | 25.98 | 69.23 | 43.25 |  | 55.69 | 43.58 | -12.12 |  | 45.83 | 43.96 | -1.87 |
|  |  | Originators | 19.83 | 10.32 | -9.51 |  | 27.71 | 25.38 | -2.33 |  | 17.96 | 22.15 | 4.20 |  | 28.62 | 28.71 | 0.08 |
| 5 | Fosinopril* | GCE uncertificated generics | 0.00 | 0.00 | 0.00 |  | 0.00 | 0.00 | 0.00 |  | 0.00 | 0.00 | 0.00 |  | 0.00 | 0.00 | 0.00 |
|  |  | GCE certificated generics | 34.43 | 3.03 | -31.41 |  | 27.86 | 5.62 | -22.24 |  | 77.96 | 79.06 | 1.10 |  | 72.09 | 74.99 | 2.91 |
|  |  | Originators | 65.57 | 96.97 | 31.41 |  | 72.14 | 94.38 | 22.24 |  | 22.04 | 20.94 | -1.10 |  | 27.91 | 25.01 | -2.91 |
| 6 | Lisinopril | GCE uncertificated generics | 95.43 | 14.11 | -81.32 |  | 91.55 | 50.80 | -40.75 |  | 59.62 | 79.87 | 20.25 |  | 61.12 | 80.37 | 19.25 |
|  |  | GCE certificated generics | 4.57 | 85.89 | 81.32 |  | 8.45 | 49.20 | 40.75 |  | 40.38 | 20.13 | -20.25 |  | 38.88 | 19.63 | -19.25 |
|  |  | Originators | 0.00 | 0.00 | 0.00 |  | 0.00 | 0.00 | 0.00 |  | 0.00 | 0.00 | 0.00 |  | 0.00 | 0.00 | 0.00 |
| 7 | Enalapril | GCE uncertificated generics | 56.71 | 10.56 | -46.15 |  | 47.90 | 13.71 | -34.19 |  | 61.18 | 54.50 | -6.69 |  | 48.47 | 43.05 | -5.42 |
|  |  | GCE certificated generics | 42.25 | 89.15 | 46.90 |  | 50.52 | 85.72 | 35.20 |  | 38.00 | 44.99 | 7.00 |  | 50.48 | 56.36 | 5.88 |
|  |  | Originators | 1.05 | 0.29 | -0.76 |  | 1.58 | 0.57 | -1.01 |  | 0.82 | 0.51 | -0.31 |  | 1.05 | 0.60 | -0.45 |
| 8 | Atorvastatin | GCE uncertificated generics | 18.38 | 0.65 | -17.73 |  | 15.71 | 1.43 | -14.29 |  | 22.02 | 21.73 | -0.30 |  | 18.99 | 18.41 | -0.57 |
|  |  | GCE certificated generics | 20.74 | 69.39 | 48.65 |  | 17.34 | 28.05 | 10.71 |  | 30.70 | 29.62 | -1.08 |  | 26.08 | 24.90 | -1.18 |
|  |  | Originators | 60.87 | 29.95 | -30.92 |  | 66.94 | 70.52 | 3.58 |  | 47.27 | 48.65 | 1.38 |  | 54.93 | 56.69 | 1.76 |
| 9 | Rosuvastatin | GCE uncertificated generics | 3.34 | 0.11 | -3.24 |  | 4.00 | 0.34 | -3.66 |  | 1.28 | 1.65 | 0.37 |  | 1.51 | 1.94 | 0.44 |
|  |  | GCE certificated generics | 59.71 | 82.15 | 22.45 |  | 48.71 | 50.78 | 2.07 |  | 60.04 | 56.36 | -3.69 |  | 49.56 | 46.36 | -3.20 |
|  |  | Originators | 36.95 | 17.74 | -19.21 |  | 47.29 | 48.88 | 1.59 |  | 38.68 | 41.99 | 3.32 |  | 48.93 | 51.69 | 2.76 |
| 10 | Levetiracetam | GCE uncertificated generics | 1.97 | 1.19 | -0.78 |  | 1.34 | 0.80 | -0.55 |  | 2.39 | 5.12 | 2.74 |  | 1.63 | 3.20 | 1.57 |
|  |  | GCE certificated generics | 3.41 | 32.99 | 29.58 |  | 3.10 | 26.14 | 23.04 |  | 4.19 | 9.19 | 5.00 |  | 3.97 | 16.27 | 12.30 |
|  |  | Originators | 94.62 | 65.82 | -28.80 |  | 95.55 | 73.06 | -22.49 |  | 93.42 | 85.69 | -7.73 |  | 94.39 | 80.53 | -13.86 |
| 11 | Olanzapine | GCE uncertificated generics | 32.72 | 15.52 | -17.21 |  | 24.15 | 13.12 | -11.03 |  | 21.39 | 29.18 | 7.80 |  | 16.51 | 21.94 | 5.44 |
|  |  | GCE certificated generics | 56.61 | 75.40 | 18.79 |  | 55.21 | 66.86 | 11.65 |  | 71.73 | 64.32 | -7.42 |  | 68.80 | 65.02 | -3.78 |
|  |  | Originators | 10.67 | 9.08 | -1.58 |  | 20.64 | 20.02 | -0.62 |  | 6.88 | 6.50 | -0.38 |  | 14.70 | 13.04 | -1.66 |
| 12 | Risperidone | GCE uncertificated generics | 43.30 | 19.57 | -23.73 |  | 30.89 | 27.64 | -3.25 |  | 51.18 | 53.93 | 2.74 |  | 49.03 | 48.72 | -0.31 |
|  |  | GCE certificated generics | 24.74 | 61.32 | 36.58 |  | 24.45 | 33.85 | 9.40 |  | 31.44 | 29.06 | -2.38 |  | 22.83 | 24.30 | 1.47 |
|  |  | Originators | 31.96 | 19.11 | -12.85 |  | 44.66 | 38.51 | -6.15 |  | 17.37 | 17.02 | -0.36 |  | 28.14 | 26.97 | -1.16 |
| 13 | Dexmedetomidine | GCE uncertificated generics | 98.43 | 16.99 | -81.44 |  | 99.35 | 16.52 | -82.83 |  | 98.60 | 85.76 | -12.85 |  | 98.70 | 83.43 | -15.26 |
|  |  | GCE certificated generics | 1.57 | 83.01 | 81.44 |  | 0.65 | 83.48 | 82.83 |  | 1.40 | 14.24 | 12.85 |  | 1.30 | 16.57 | 15.26 |
|  |  | Originators | 0.00 | 0.00 | 0.00 |  | 0.00 | 0.00 | 0.00 |  | 0.00 | 0.00 | 0.00 |  | 0.00 | 0.00 | 0.00 |
| 14 | Escitalopram | GCE uncertificated generics | 2.75 | 0.59 | -2.16 |  | 1.46 | 0.39 | -1.07 |  | 2.37 | 3.69 | 1.32 |  | 2.01 | 3.17 | 1.16 |
|  |  | GCE certificated generics | 50.10 | 72.91 | 22.81 |  | 38.40 | 54.90 | 16.51 |  | 72.10 | 73.99 | 1.90 |  | 58.45 | 62.58 | 4.13 |
|  |  | Originators | 47.15 | 26.50 | -20.65 |  | 60.14 | 44.71 | -15.43 |  | 25.53 | 22.32 | -3.21 |  | 39.55 | 34.25 | -5.30 |
| 15 | Paroxetine | GCE uncertificated generics | 45.45 | 13.07 | -32.39 |  | 54.21 | 26.92 | -27.29 |  | 43.76 | 44.91 | 1.16 |  | 49.41 | 43.65 | -5.77 |
|  |  | GCE certificated generics | 54.55 | 86.93 | 32.39 |  | 45.79 | 73.08 | 27.29 |  | 56.24 | 55.09 | -1.16 |  | 50.59 | 56.35 | 5.77 |
|  |  | Originators | 0.00 | 0.00 | 0.00 |  | 0.00 | 0.00 | 0.00 |  | 0.00 | 0.00 | 0.00 |  | 0.00 | 0.00 | 0.00 |
| 16 | Gefitinib* | GCE uncertificated generics | 0.00 | 0.02 | 0.02 |  | 0.00 | 0.01 | 0.01 |  | 0.00 | 0.01 | 0.01 |  | 0.00 | 0.00 | 0.00 |
|  |  | GCE certificated generics | 23.91 | 16.50 | -7.41 |  | 14.74 | 16.53 | 1.78 |  | 23.28 | 58.21 | 34.93 |  | 16.31 | 36.39 | 20.08 |
|  |  | Originators | 76.09 | 83.48 | 7.39 |  | 85.26 | 83.46 | -1.80 |  | 76.72 | 41.78 | -34.94 |  | 83.69 | 63.61 | -20.08 |
| 17 | Imatinib | GCE uncertificated generics | 50.11 | 14.31 | -35.80 |  | 17.20 | 4.72 | -12.48 |  | 24.97 | 22.82 | -2.15 |  | 7.37 | 7.60 | 0.23 |
|  |  | GCE certificated generics | 31.27 | 67.95 | 36.67 |  | 12.58 | 23.44 | 10.86 |  | 54.85 | 57.91 | 3.06 |  | 19.63 | 21.26 | 1.63 |
|  |  | Originators | 18.61 | 17.74 | -0.87 |  | 70.22 | 71.84 | 1.62 |  | 20.19 | 19.27 | -0.91 |  | 73.00 | 71.14 | -1.86 |
| 18 | Pemetrexed | GCE uncertificated generics | 90.69 | 49.68 | -41.01 |  | 79.13 | 45.71 | -33.42 |  | 97.57 | 95.12 | -2.45 |  | 93.54 | 88.46 | -5.09 |
|  |  | GCE certificated generics | 0.29 | 42.99 | 42.71 |  | 0.35 | 37.31 | 36.97 |  | 0.13 | 2.09 | 1.95 |  | 0.32 | 4.68 | 4.36 |
|  |  | Originators | 9.03 | 7.33 | -1.70 |  | 20.53 | 16.98 | -3.55 |  | 2.30 | 2.80 | 0.50 |  | 6.14 | 6.86 | 0.72 |
| 19 | Cefuroxime | GCE uncertificated generics | 24.25 | 7.71 | -16.54 |  | 32.31 | 18.47 | -13.84 |  | 31.73 | 29.34 | -2.40 |  | 26.09 | 24.99 | -1.10 |
|  |  | GCE certificated generics | 72.45 | 90.24 | 17.79 |  | 62.99 | 76.41 | 13.41 |  | 60.90 | 62.38 | 1.48 |  | 65.23 | 65.65 | 0.43 |
|  |  | Originators | 3.30 | 2.05 | -1.25 |  | 4.70 | 5.12 | 0.42 |  | 7.37 | 8.29 | 0.92 |  | 8.68 | 9.36 | 0.68 |
| 20 | Entecavir | GCE uncertificated generics | 6.27 | 0.13 | -6.15 |  | 4.27 | 0.41 | -3.86 |  | 5.71 | 6.86 | 1.15 |  | 4.52 | 5.57 | 1.05 |
|  |  | GCE certificated generics | 70.21 | 90.25 | 20.04 |  | 51.92 | 31.13 | -20.79 |  | 82.88 | 83.86 | 0.99 |  | 71.72 | 73.44 | 1.72 |
|  |  | Originators | 23.51 | 9.62 | -13.89 |  | 43.81 | 68.46 | 24.65 |  | 11.41 | 9.28 | -2.13 |  | 23.76 | 20.99 | -2.77 |
| 21 | Tenofovir Disoproxil | GCE uncertificated generics | 0.00 | 0.00 | 0.00 |  | 0.00 | 0.00 | 0.00 |  | 0.00 | 0.00 | 0.00 |  | 0.00 | 0.00 | 0.00 |
|  |  | GCE certificated generics | 30.79 | 78.95 | 48.16 |  | 29.60 | 43.31 | 13.71 |  | 33.06 | 72.44 | 39.38 |  | 31.39 | 70.28 | 38.89 |
|  |  | Originators | 69.21 | 21.05 | -48.16 |  | 70.40 | 56.69 | -13.71 |  | 66.94 | 27.56 | -39.38 |  | 68.61 | 29.72 | -38.89 |
| 22 | Montmorillonite | GCE uncertificated generics | 63.76 | 19.33 | -44.43 |  | 27.39 | 12.88 | -14.51 |  | 60.04 | 46.33 | -13.71 |  | 44.80 | 38.45 | -6.35 |
|  |  | GCE certificated generics | 8.95 | 73.75 | 64.80 |  | 10.77 | 71.92 | 61.15 |  | 17.61 | 29.99 | 12.38 |  | 14.05 | 24.54 | 10.49 |
|  |  | Originators | 27.29 | 6.92 | -20.37 |  | 61.84 | 15.20 | -46.65 |  | 22.35 | 23.68 | 1.33 |  | 41.15 | 37.02 | -4.14 |
| 23 | Clopidogrel | GCE uncertificated generics | 0.00 | 0.00 | 0.00 |  | 0.00 | 0.00 | 0.00 |  | 0.00 | 0.00 | 0.00 |  | 0.00 | 0.00 | 0.00 |
|  |  | GCE certificated generics | 58.86 | 78.53 | 19.67 |  | 44.93 | 51.22 | 6.29 |  | 56.03 | 54.05 | -1.98 |  | 42.13 | 40.10 | -2.03 |
|  |  | Originators | 41.14 | 21.47 | -19.67 |  | 55.07 | 48.78 | -6.29 |  | 43.97 | 45.95 | 1.98 |  | 57.87 | 59.90 | 2.03 |
| 24 | Flurbiprofen* | GCE uncertificated generics | 0.00 | 0.00 | 0.00 |  | 0.00 | 0.00 | 0.00 |  | 0.00 | 0.00 | 0.00 |  | 0.00 | 0.00 | 0.00 |
|  |  | GCE certificated generics | 3.27 | 6.08 | 2.81 |  | 0.73 | 4.26 | 3.53 |  | 1.34 | 15.97 | 14.63 |  | 1.20 | 16.29 | 15.09 |
|  |  | Originators | 96.73 | 93.92 | -2.81 |  | 99.27 | 95.74 | -3.53 |  | 98.66 | 84.03 | -14.63 |  | 98.80 | 83.71 | -15.09 |
| 25 | Montelukast | GCE uncertificated generics | 39.19 | 12.89 | -26.30 |  | 33.51 | 15.83 | -17.67 |  | 33.54 | 35.25 | 1.72 |  | 30.77 | 31.68 | 0.91 |
|  |  | GCE certificated generics | 14.10 | 69.99 | 55.89 |  | 26.33 | 69.14 | 42.80 |  | 21.15 | 23.96 | 2.81 |  | 34.79 | 38.60 | 3.81 |
|  |  | Originators | 46.72 | 17.12 | -29.60 |  | 40.16 | 15.03 | -25.13 |  | 45.31 | 40.79 | -4.53 |  | 34.43 | 29.72 | -4.72 |
|  | Total | GCE uncertificated generics | 24.63 | 2.71 | -21.92 |  | 20.33 | 8.03 | -12.30 |  | 31.65 | 28.70 | -2.95 |  | 22.66 | 21.75 | -0.90 |
|  |  | GCE certificated generics | 35.14 | 75.90 | 40.76 |  | 26.95 | 43.85 | 16.90 |  | 42.14 | 42.39 | 0.25 |  | 35.54 | 37.05 | 1.51 |
|  |  | Originators | 40.23 | 21.38 | -18.85 |  | 52.72 | 48.12 | -4.60 |  | 26.21 | 28.91 | 2.70 |  | 41.80 | 41.20 | -0.60 |

* indicates that original brand-name enterprise won the bid. GCE, generic consistency evaluation; NVBP, national volume-based procurement; △ refers to the difference value between post- and pre-NVBP periods.

# Appendix D. Full results of seller concentration analysis

## **Table A.** Descriptive changes in HHI-volume before and after NVBP policy in pilot and control regions.

| No. | Name of drug ingredient | Pilot regions | | |  | Control regions | | |
| --- | --- | --- | --- | --- | --- | --- | --- | --- |
|  |  | Pre- NVBP | Post- NVBP | △ |  | Pre- NVBP | Post- NVBP | △ |
| 1 | Amlodipine | 2518.68 | 4976.07 | 2457.39 |  | 1231.34 | 1276.54 | 45.20 |
| 2 | Losartan | 3278.89 | 6396.34 | 3117.45 |  | 2126.32 | 1828.36 | -297.96 |
| 3 | Irbesartan | 1880.60 | 6237.53 | 4356.93 |  | 1336.81 | 1662.61 | 325.80 |
| 4 | Irbesartan and  Hydrochlorothiazide | 1779.03 | 6793.08 | 5014.05 |  | 2150.59 | 2091.41 | -59.18 |
| 5 | Fosinopril | 5484.58 | 9413.02 | 3928.44 |  | 6563.39 | 6689.09 | 125.70 |
| 6 | Lisinopril | 3527.69 | 7530.69 | 4003.00 |  | 2630.27 | 3045.49 | 415.22 |
| 7 | Enalapril | 2477.58 | 7971.90 | 5494.32 |  | 1855.20 | 2345.94 | 490.74 |
| 8 | Atorvastatin | 3538.86 | 5460.82 | 1921.96 |  | 3160.37 | 3179.39 | 19.02 |
| 9 | Rosuvastatin | 2489.86 | 5830.03 | 3340.17 |  | 2424.69 | 2495.01 | 70.32 |
| 10 | Levetiracetam | 8963.02 | 5383.74 | -3579.28 |  | 8741.81 | 7411.28 | -1330.53 |
| 11 | Olanzapine | 3877.79 | 5922.06 | 2044.27 |  | 5595.03 | 4842.22 | -752.81 |
| 12 | Risperidone | 1972.20 | 3658.45 | 1686.25 |  | 1651.25 | 1623.05 | -28.20 |
| 13 | Dexmedetomidine | 5376.81 | 7066.11 | 1689.30 |  | 7301.07 | 5159.57 | -2141.50 |
| 14 | Escitalopram | 3755.66 | 4066.33 | 310.67 |  | 2793.90 | 2602.48 | -191.42 |
| 15 | Paroxetine | 3436.20 | 6377.02 | 2940.82 |  | 3861.50 | 3340.94 | -520.56 |
| 16 | Gefitinib | 6361.44 | 7241.90 | 880.46 |  | 6427.69 | 5133.98 | -1293.71 |
| 17 | Imatinib | 3683.88 | 5115.15 | 1431.27 |  | 3884.60 | 4047.18 | 162.58 |
| 18 | Pemetrexed | 2380.48 | 2732.27 | 351.79 |  | 3268.75 | 2751.94 | -516.81 |
| 19 | Cefuroxime | 1588.54 | 6091.58 | 4503.04 |  | 2526.14 | 2587.53 | 61.39 |
| 20 | Entecavir | 2988.72 | 7812.79 | 4824.07 |  | 3215.78 | 3024.06 | -191.72 |
| 21 | Tenofovir Disoproxil | 5358.02 | 6505.73 | 1147.71 |  | 4909.90 | 2995.48 | -1914.42 |
| 22 | Montmorillonite | 2243.11 | 5468.24 | 3225.13 |  | 1448.16 | 1595.68 | 147.52 |
| 23 | Clopidogrel | 3525.27 | 6160.30 | 2635.03 |  | 3692.32 | 3670.76 | -21.56 |
| 24 | Flurbiprofen | 9367.53 | 8858.20 | -509.33 |  | 9735.24 | 7315.49 | -2419.75 |
| 25 | Montelukast | 4424.94 | 3865.80 | -559.14 |  | 5013.12 | 4462.39 | -550.73 |
|  | Average | 3851.18 | 6117.41 | 2266.23 |  | 3901.81 | 3487.11 | -414.70 |

NVBP, national volume-based procurement; △ refers to the difference value between post- and pre-NVBP periods.

## **Table B.** Descriptive changes in HHI-value before and after NVBP policy in pilot and control regions.

| No. | Name of drug ingredient | Pilot regions | | |  | Control regions | | |
| --- | --- | --- | --- | --- | --- | --- | --- | --- |
|  |  | Pre- NVBP | Post- NVBP | △ |  | Pre- NVBP | Post- NVBP | △ |
| 1 | Amlodipine | 5317.69 | 6374.14 | 1056.45 |  | 2871.39 | 2893.76 | 22.37 |
| 2 | Losartan | 3693.87 | 4240.12 | 546.25 |  | 2010.15 | 1796.89 | -213.26 |
| 3 | Irbesartan | 2886.87 | 4597.85 | 1710.98 |  | 1910.60 | 2184.76 | 274.16 |
| 4 | Irbesartan and  Hydrochlorothiazide | 1901.17 | 4613.28 | 2712.11 |  | 2175.44 | 2270.67 | 95.23 |
| 5 | Fosinopril | 5980.53 | 8939.64 | 2959.11 |  | 5975.65 | 6249.28 | 273.63 |
| 6 | Lisinopril | 2596.46 | 4593.36 | 1996.90 |  | 2735.50 | 3635.22 | 899.72 |
| 7 | Enalapril | 3110.86 | 7391.31 | 4280.45 |  | 2906.73 | 3406.21 | 499.48 |
| 8 | Atorvastatin | 4033.63 | 5169.93 | 1136.30 |  | 3687.81 | 3810.94 | 123.13 |
| 9 | Rosuvastatin | 3085.64 | 3606.43 | 520.79 |  | 3070.06 | 3215.70 | 145.64 |
| 10 | Levetiracetam | 9136.63 | 5941.86 | -3194.77 |  | 8920.44 | 6600.63 | -2319.81 |
| 11 | Olanzapine | 3719.07 | 4805.34 | 1086.27 |  | 5186.23 | 4755.35 | -430.88 |
| 12 | Risperidone | 3724.84 | 3945.56 | 220.72 |  | 1976.19 | 1874.60 | -101.59 |
| 13 | Dexmedetomidine | 5498.99 | 7147.21 | 1648.22 |  | 7426.82 | 5555.97 | -1870.85 |
| 14 | Escitalopram | 4574.58 | 3595.35 | -979.23 |  | 3115.33 | 2849.23 | -266.10 |
| 15 | Paroxetine | 3383.28 | 3741.68 | 358.40 |  | 3432.15 | 3260.99 | -171.16 |
| 16 | Gefitinib | 7485.88 | 7238.40 | -247.48 |  | 7270.08 | 5370.13 | -1899.95 |
| 17 | Imatinib | 5366.51 | 5729.83 | 363.32 |  | 5757.02 | 5549.68 | -207.34 |
| 18 | Pemetrexed | 2349.17 | 2391.27 | 42.10 |  | 3771.58 | 2997.00 | -774.58 |
| 19 | Cefuroxime | 1774.47 | 2725.91 | 951.44 |  | 2815.87 | 2739.02 | -76.85 |
| 20 | Entecavir | 3604.60 | 5337.46 | 1732.86 |  | 3064.13 | 2765.97 | -298.16 |
| 21 | Tenofovir Disoproxil | 5492.89 | 4881.59 | -611.30 |  | 5102.95 | 3138.03 | -1964.92 |
| 22 | Montmorillonite | 4099.08 | 5325.44 | 1226.36 |  | 2206.35 | 1997.34 | -209.01 |
| 23 | Clopidogrel | 4192.27 | 4749.33 | 557.06 |  | 4446.10 | 4519.82 | 73.72 |
| 24 | Flurbiprofen | 9855.19 | 9184.43 | -670.76 |  | 9762.59 | 7272.62 | -2489.97 |
| 25 | Montelukast | 4858.35 | 3510.01 | -1348.34 |  | 5230.27 | 4405.48 | -824.79 |
|  | Average | 4468.90 | 5191.07 | 722.17 |  | 4273.10 | 3804.61 | -468.49 |

NVBP, national volume-based procurement; △ refers to the difference value between post- and pre-NVBP periods.

## **Figure A.** Results of parallel trend test for DID estimation models.

*(1) The overall results*


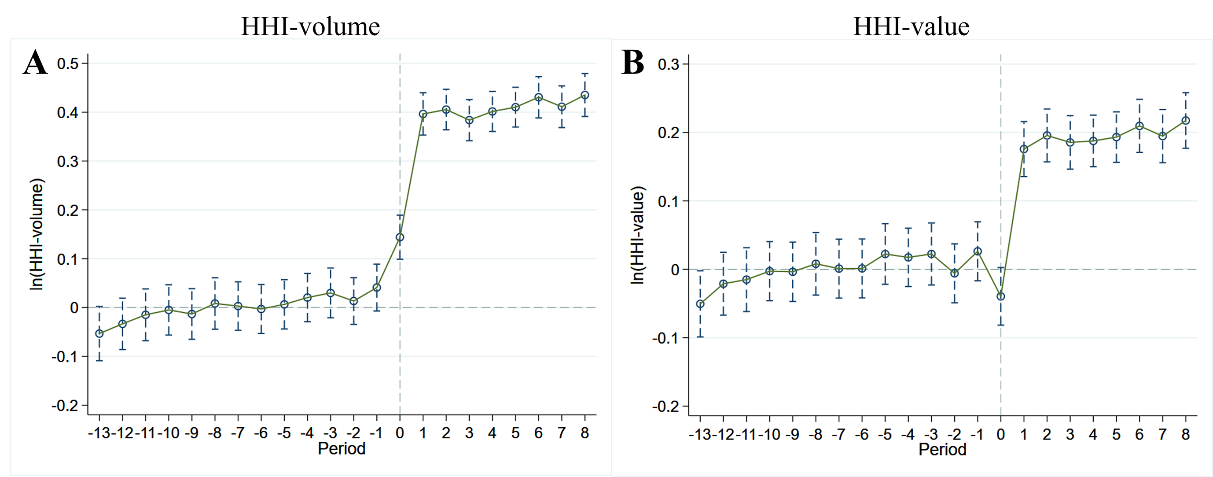


*(2) Sub-group analysis by bid-winner’s baseline market share level: A,B-low market share; C,D-medium market share; E,F-high market share.*


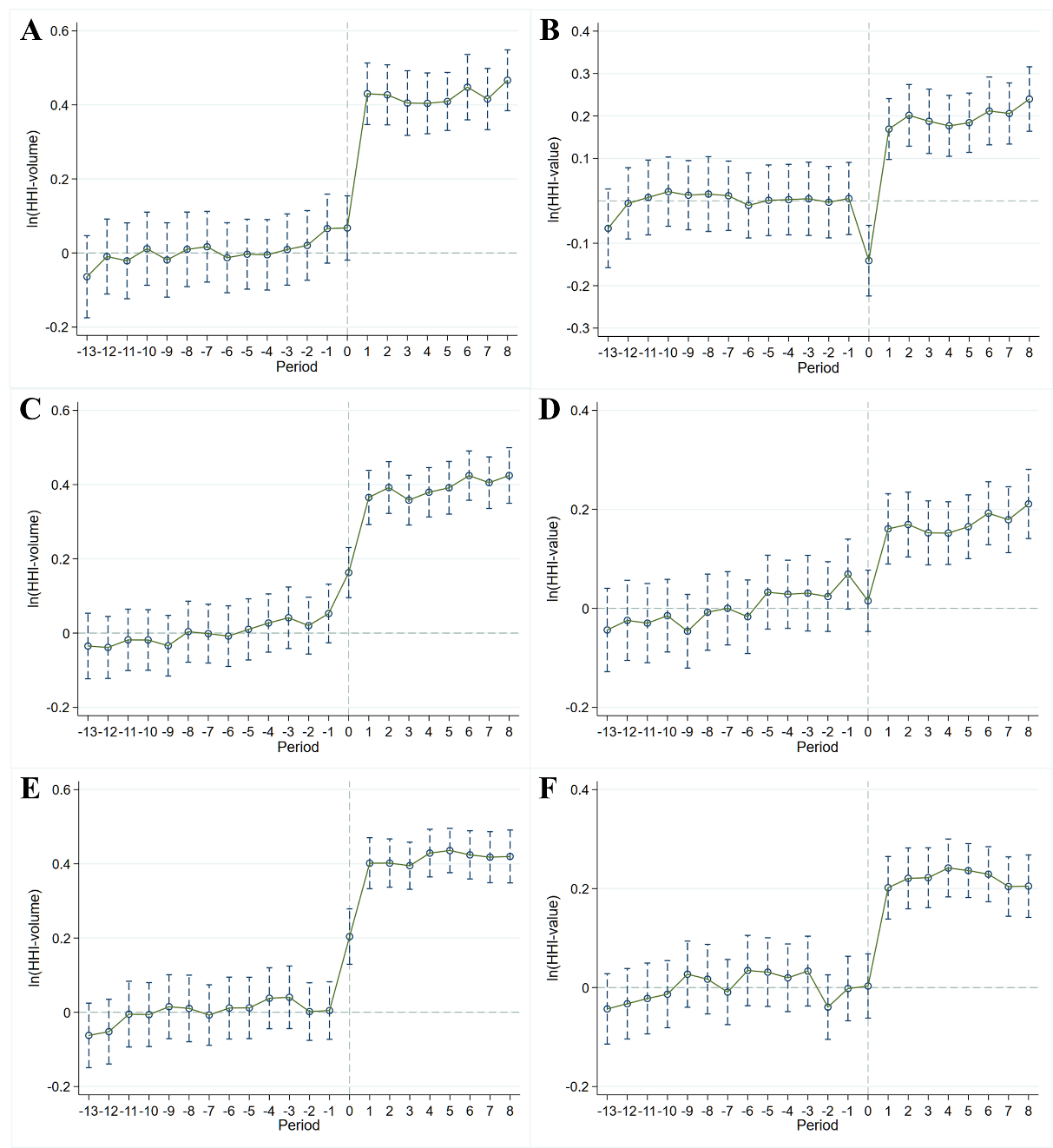


*(3) Sub-group analysis by bid-winning enterprise’s characteristic: A,B-originators won the bid; C,D-* *generics won the bid.*


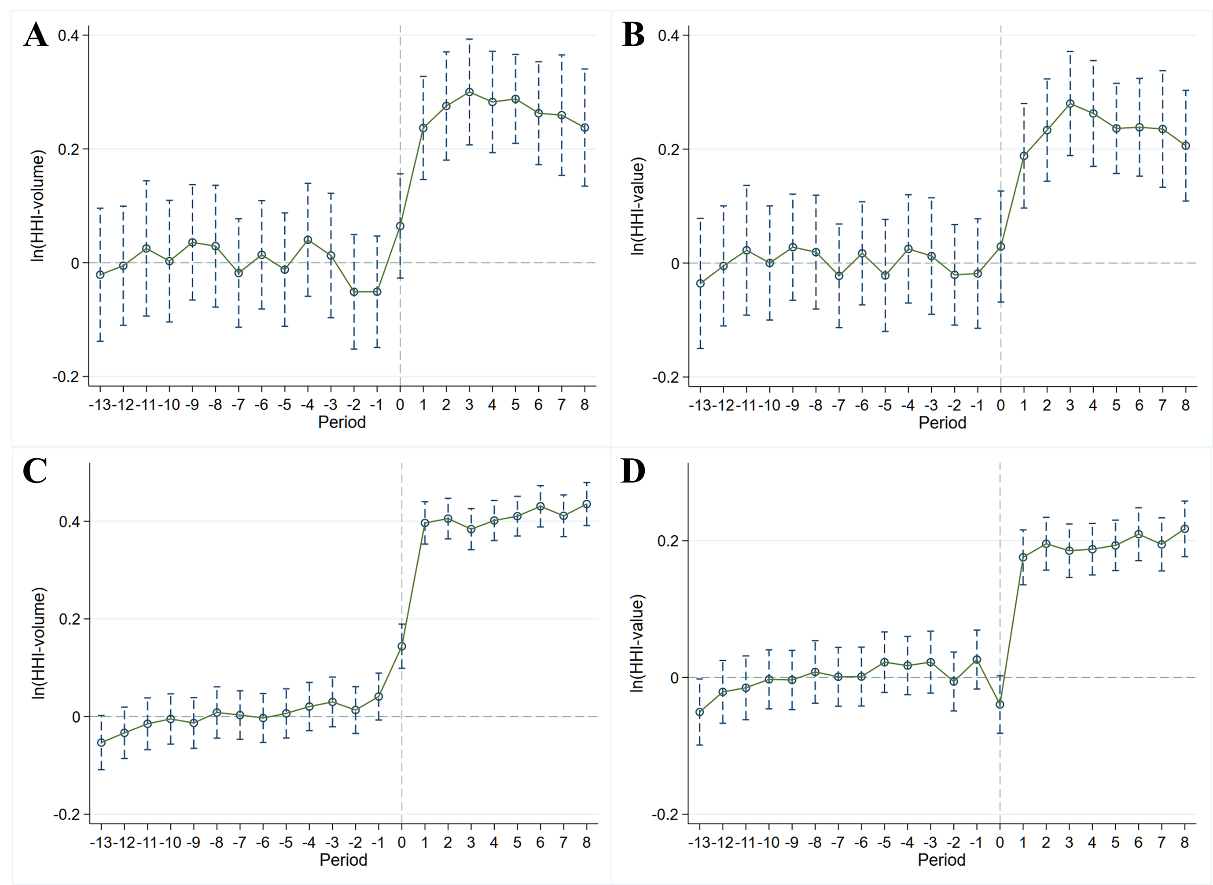


1. According to the National Bureau of Statistics, Chinese mainland can be divided into seven geographical regions - Eastern China, North China, South China, Central China, Northeast China, Southwest China, and Northwest China. Considering the feasibility of pilot city classification, this study combined the Central China and South China into the Central and Southern China. [↑](#footnote-ref-0)
2. The eleven included cities are 4 municipalities and 7 sub-provincial cities, belonging to relatively developed regions in Chinese mainland. Strictly speaking, it is almost impossible to find regions that are exactly equal to the pilot cities in terms of population, society, economy, and medical in Chinese mainland. [↑](#footnote-ref-1)
